# RBM15/IGF2BP2–PTPRH m6A regulatory axis in non-small cell lung cancer

**DOI:** 10.1007/s13402-026-01217-2

**Published:** 2026-05-11

**Authors:** Keyue Qiu, Xiaoxiao Zheng, Hongxiang Li, Jiaheng Zhang, Zijun Xie, Hang Chen, Zeyang Hu, Yiyi Sun, Zhe Chen, Jingtao Tong, Yinyu Mu, Yuanyuan Yao, Wei Chen, Guodong Xu

**Affiliations:** 1 Department of Thoracic Surgery, The Affiliated Lihuili Hospital of Ningbo University, Ningbo, Zhejiang 315000 China; 2 Health Science Center, Ningbo University, Ningbo, Zhejiang 315211 China; 3 Cancer Institute of Integrated Traditional Chinese and Western Medicine, Key Laboratory of Cancer Prevention and Therapy Combining Traditional Chinese and Western Medicine of Zhejiang Province, Zhejiang Academy of Traditional Chinese Medicine, Tongde Hospital of Zhejiang Province, Hangzhou, Zhejiang, 310012 China; 4 The Third People’s Hospital Health Care Group of Cixi, Ningbo, Zhejiang 315300 China; 5 Department of Tumor Radiotherapy and Chemotherapy Center, The Affiliated Lihuili Hospital of Ningbo University, Ningbo, Zhejiang 315000 China; 6 Department of Clinical Laboratory, The Affiliated Lihuili Hospital of Ningbo University, Ningbo, Zhejiang 315000 China; 7 Yuyao Hospital of Traditional Chinese Medicine, Ningbo, Zhejiang 315400 China; 8 Department of General Surgery, Sir Run Run Shaw Hospital, School of Medicine, Zhejiang University, Hangzhou, Zhejiang, 310016 China; 9 Key Laboratory for Accurate Diagnosis and Treatment of Abdominal Infection in Zhejiang Province, Sir Run Run Shaw Hospital, School of Medicine, Zhejiang University, Hangzhou, Zhejiang, 310016 China

**Keywords:** Non-small cell lung cancer (NSCLC), N6-methyladenosine (m6A), PTPRH, RBM15, IGF2BP2

## Abstract

**Background:**

Non-small cell lung cancer (NSCLC) remains a leading cause of cancer-related death globally, highlighting the urgent need for new molecular drivers and therapeutic targets. This study investigates the role of protein tyrosine phosphatase receptor type H (PTPRH) and its regulation through an epitranscriptomic mechanism in NSCLC.

**Methods:**

We performed an integrated analysis of datasets from The Cancer Genome Atlas and Gene Expression Omnibus to evaluate PTPRH expression and its prognostic significance. PTPRH levels were further validated in clinical NSCLC specimens and correlated with clinicopathological features. We assessed the functional consequences of PTPRH expression on proliferation, apoptosis, migration, invasion, and angiogenesis using in vitro and in vivo models. The underlying mechanism involving N6-methyladenosine (m6A) modification was explored by examining the roles of the m6A writer RBM15 and the reader IGF2BP2 on PTPRH transcript stability. Gain- and loss-of-function experiments, coupled with rescue studies, were conducted to delineate the functional axis.

**Results:**

PTPRH was significantly overexpressed in NSCLC tissues, and its elevated expression correlated with poor patient prognosis. Functionally, PTPRH promoted tumor cell proliferation, migration, invasion, and xenograft tumor growth, while simultaneously inhibiting apoptosis and enhancing angiogenesis via the VEGF pathway. Mechanistically, PTPRH expression was post-transcriptionally stabilized by m6A methylation. RBM15 facilitated m6A deposition on PTPRH transcripts, while IGF2BP2 binding to these modified sites enhanced PTPRH mRNA stability. Depletion of RBM15 or IGF2BP2 reduced PTPRH levels and suppressed malignant behaviors, whereas their overexpression produced the opposite effects. The oncogenic functions of PTPRH were confirmed to be dependent on this regulatory axis.

**Conclusion:**

Our study unveils a novel epigenetic regulatory axis in which RBM15-mediated m6A modification and IGF2BP2-dependent recognition stabilize PTPRH mRNA, thereby promoting NSCLC progression. This work expands the understanding of post-transcriptional regulation in lung cancer and identifies the RBM15/IGF2BP2–PTPRH axis as a potential therapeutic target for NSCLC intervention.

**Supplementary Information:**

The online version contains supplementary material available at 10.1007/s13402-026-01217-2.

## Introduction

Lung cancer remains one of the most aggressive malignancies, with the highest incidence and mortality rates worldwide, posing a major threat to public health [1]. According to the most recent global cancer statistics, both incidence and mortality rank first among all cancers [2]. Non-small cell lung cancer (NSCLC) accounts for approximately 85% of lung cancer cases, with lung adenocarcinoma (LUAD) representing nearly half [3]. Despite advances in diagnosis and treatment, almost 80% of patients are diagnosed at an advanced stage [4].

Accumulating evidence has revealed that epigenetic modifications critically shape tumor biology, influencing proliferation, immune evasion, and metastasis. Dysregulated epigenetic mechanisms have been shown to profoundly impact cancer development and therapeutic response [5], highlighting epigenetically mediated processes as promising targets for NSCLC therapy.

Among these mechanisms, N6-methyladenosine (m6A) is the most abundant internal modification in eukaryotic mRNA and regulates mRNA splicing, stability, and translation [6]. Aberrations in m6A “writers,” “erasers,” and “readers” have been linked to tumorigenesis, progression, and prognosis in multiple cancer types, including pancreatic [7], ovarian [8], lung [9], liver [10], and breast cancers [11]. As part of the methyltransferase complex, RNA-binding motif protein 15 (RBM15) recruits the m6A machinery to specific RNA sites [12, 13]. RBM15 has been shown to promote oncogenesis in several solid tumors [14, 15], and our previous work demonstrated that it is highly expressed in NSCLC and drives osimertinib resistance via an m6A-dependent mechanism [16]. In parallel, the m6A reader insulin-like growth factor 2 mRNA-binding protein 2 (IGF2BP2) recognizes m6A-modified transcripts and enhances their stability, thereby promoting gene expression [17].

Protein tyrosine phosphatases (PTPs), together with receptor tyrosine kinases (RTKs), orchestrate phosphorylation–dephosphorylation balance and regulate diverse signaling pathways [18, 19]. PTPs are increasingly recognized as crucial players in tumorigenesis [20]. Among them, protein tyrosine phosphatase receptor type H (PTPRH), also known as stomach cancer-associated PTP-1 (SAP-1), is one of the most frequently mutated PTPs [21]. First identified in 1994 by Matozaki et al. for its high expression in pancreatic and colorectal cancer cell lines [22], PTPRH has since been implicated in regulating proliferation, migration, and invasion in multiple cancers [23–25]. Recent studies suggest a role for PTPRH in NSCLC, where its high expression correlates with poor prognosis [26] and enhances glycolysis via PI3K/AKT/mTOR signaling [27]. However, the precise regulatory mechanisms underlying PTPRH dysregulation in NSCLC remain poorly understood.

In this study, we employed integrative genomic analyses to identify novel therapeutic targets in NSCLC. By intersecting hypermethylated m6A transcripts with genes upregulated in the TCGA-LUAD cohort, PTPRH emerged as a strong candidate. We demonstrate that PTPRH is overexpressed in NSCLC, promotes malignant progression, and is regulated by RBM15- and IGF2BP2-mediated m6A stabilization. Our findings identify PTPRH as a clinically relevant oncogene and uncover an RBM15/IGF2BP2–PTPRH regulatory axis, providing mechanistic insight and potential therapeutic implications in NSCLC.

## Materials and methods

### Bioinformatics analyses

RNA-seq data from The Cancer Genome Atlas (TCGA; http://portal.gdc.cancer.gov/projects/TCGA-LUAD) were analyzed to characterize the expression profiles of *PTPRH*, *RBM15*, and *IGF2BP2* in LUAD. A total of 600 samples processed using the HTSeq-FPKM and HTSeq-Counts pipelines were included, from which gene expression data for *PTPRH*, *RBM15*, and *IGF2BP2* were extracted. Samples lacking matched RNA-seq and corresponding clinical information were excluded to ensure data integrity. Differential expression analysis between tumor and adjacent normal tissues was conducted using raw read counts (HTSeq-Counts) with the “edgeR” package (version 4.8.1) implemented in R (version 4.4.0). Genes with a false discovery rate (FDR) <0.05, based on the Benjamini–Hochberg correction, were considered significantly differentially expressed. Effect sizes are presented as log2 fold change with corresponding 95% confidence intervals. For independent validation, the GEO dataset GSE32863 (GEO Accession viewer) was analyzed.

For Kaplan–Meier survival analysis, survival data from NSCLC patients in the TCGA-LUAD cohort were evaluated. Patients were stratified into high- and low-expression groups according to the median expression levels of *PTPRH*, *RBM15*, or *IGF2BP2*. Kaplan–Meier survival curves were generated and compared using the log-rank test with the survival package in R (version 4.4.0). Hazard ratios (HRs) and 95% confidence intervals (CIs) were estimated using a univariate Cox proportional hazards model. Final survival plots, including HR (95% CI) values and risk tables, were generated using the survminer package.

Potential m6A modification sites within the PTPRH transcript were predicted using the SRAMP tool (Welcome to SRAMP, an online m6A site predictor ). Analyses were performed using the full-length transcript sequence under the “high-resolution” prediction mode, which integrates both sequence and RNA structural features. Predicted sites with high confidence scores, as defined by the algorithm, were selected for subsequent validation.

Functional enrichment was assessed using Gene Set Enrichment Analysis (GSEA, version 4.3.3; Broad Institute, Cambridge, MA, USA), while Gene Ontology (GO) analysis was performed in the TCGA cohort. Significance thresholds were set according to GSEA standards (FDR < 0.25 and nominal *p* < 0.05).

### Patient specimens

Paired LUAD tissues and adjacent non-tumorous tissues (>1 cm from the tumor margin) were obtained from 50 patients (25 males and 25 females; mean age, 65.3 ± 11.2 years) who underwent surgical resection at Ningbo Medical Center Li Huili Hospital between January and December 2024. Inclusion criteria were: (1) age ≥ 18 years; (2) histopathological confirmation of primary LUAD; and (3) provision of written informed consent. Exclusion criteria were: (1) a history of other malignancies; (2) prior receipt of radiotherapy, chemotherapy, or other anti-cancer treatments before surgery; and (3) concurrent pulmonary diseases, including tuberculosis or active inflammation. All tissue specimens were immediately snap-frozen in liquid nitrogen and stored for subsequent reverse transcription–quantitative PCR (RT-qPCR) and immunohistochemistry. The study protocol was reviewed and approved by the Institutional Ethical Review Board of Ningbo Medical Center Li Huili Hospital (Approval No. 202402042206000066192).

### Cell culture

The human lung cancer cell lines A549, PC9, H1650, H1299, H1975, HCC827, and H520; the human embryonic lung fibroblast cell line MRC-5; and human umbilical vein endothelial cells (HUVECs) were obtained from Procell (Wuhan, China). Lung cancer cell lines were cultured in Roswell Park Memorial Institute 1640 medium (RPMI-1640; Gibco, Grand Island, NY, USA). MRC-5 cells were maintained in Dulbecco’s Modified Eagle Medium (DMEM; Gibco) supplemented with 10% fetal bovine serum (FBS; Procell; Cat. No. 164,210-50) and 1% penicillin–streptomycin solution (100×; Biosharp, Hefei, China; Cat. No. BL505A). HUVECs were cultured in their specific growth medium (PUMC-HUVEC-T1; Gibco). All cell lines were authenticated by short tandem repeat profiling (≥85% match to ATCC/DSMZ reference standards) and confirmed to be free of mycoplasma contamination by monthly PCR testing (MycoAlert; Lonza, Basel, Switzerland). Cells were maintained at 37 °C in a humidified incubator with 5% CO_2_.

### Antibodies and reagents

Rabbit anti-PTPRH antibody (ab231767; Abcam, Shanghai, China) was used for protein detection. Rabbit anti-RBM15 (#60386), rabbit anti-PARP (#9532), rabbit anti–cleaved PARP (#9541), rabbit anti-caspase-3 (#9662), rabbit anti-BAX (#2772), rabbit anti-BCL-2 (#3498), rabbit anti-HIF-1α (#14179), rabbit anti-β-Tubulin (# 2146), and rabbit anti-GAPDH (#2118) antibodies were obtained from Cell Signaling Technology (Danvers, MA, USA). Rabbit anti-IGF2BP2 (11601–1-AP) and rabbit anti–cleaved caspase-3 (25128–1-AP) were purchased from Proteintech (Wuhan, China). Mouse anti-VEGF (sc-53462) was purchased from Santa Cruz Biotechnology (Dallas, TX, USA). For Western blot analysis, primary antibodies were diluted at 1:1000, and secondary antibodies were diluted at 1:2000.

### Lentiviral construction and transfection, and small interfering RNA (siRNA)

Lentiviral systems for gene overexpression or knockdown were purchased from Shanghai GenePharma (Shanghai, China), with vectors carrying a puromycin resistance marker. For transduction, HCC827 and H1975 cells were seeded in 6-well plates at 60% confluence, and lentiviral suspensions were added at a multiplicity of infection (MOI) of 10 with 5 μg/mL Polybrene (GenePharma). After overnight incubation, the virus–Polybrene mixture was replaced with fresh complete medium. Following 48 h of culture, cells were selected with 2 μg/mL puromycin (MedChemExpress, Monmouth Junction, NJ, USA; Cat. No. HY-K1057) for 72 h to establish stable polyclonal populations. Small interfering RNAs were synthesized by Shanghai GenePharma and transfected into cells using Lipofectamine 2000 (Invitrogen, Waltham, MA, USA; Cat. No. 11668019). Cells were harvested 48 h post-transfection for subsequent analyses. Gene overexpression or knockdown efficiency was confirmed by Western blot analysis and RT-qPCR. The shRNA and siRNA sequences are provided in Supplementary Table S1.

### Reverse transcription quantitative real-time PCR (RT-qPCR)

Total RNA was extracted from cultured cells using RNAiso Plus (Takara Bio, Kusatsu, Japan; Cat. No. 9109). RNA concentration was measured at 260 nm using a NanoDrop 2000 spectrophotometer (Thermo Fisher Scientific, Wilmington, DE, USA). First-strand cDNA was synthesized with the PrimeScript™ RT Reagent Kit (Takara Bio; Cat. No. RR047A). Quantitative real-time PCR was performed using TB Green® Premix Ex Taq™ II (Takara Bio; Cat. No. RR420A) on a QuantStudio™ 5 System (Applied Biosystems, Singapore). β-Actin (ACTB) was used as the endogenous control. Relative gene expression was calculated using the 2^−ΔΔCT^ method, normalized to the reference group. All reactions were performed in triplicate. Primer sequences are provided in Supplementary Table S1.

### Western blot analysis

Cells were lysed in ice-cold RIPA buffer (SolarBio, Beijing, China; Cat. No. R0010) supplemented with protease inhibitors (Roche, Shanghai, China; Cat. No. 04693116001) and phosphatase inhibitors (Absin, Shanghai, China; Cat. No. 725A029). Protein concentrations were measured using the BCA Protein Assay Kit (Applygen, Beijing, China; Cat. No. P1511). Equal amounts of protein lysates (20 μg per lane) were resolved by SDS-PAGE (10% gels for most proteins, 8% for PTPRH, and 15% for BAX, BCL-2, and cleaved caspase-3) and transferred to the Immobilon®-P PVDF membranes (MilliporeSigma, Merck KGaA, Darmstadt, Germany; Cat. No. IPVH00010). Membranes were blocked with 5% (w/v) non-fat milk in TBST (Tris-buffered saline with 0.1% Tween-20) for 1 h at room temperature, followed by overnight incubation at 4 °C with primary antibodies diluted in TBST. After three 10-min washes, membranes were incubated with horseradish peroxidase (HRP)-conjugated secondary antibodies (Cell Signaling Technology, Danvers, MA, USA; Cat. No. 7074S; 1:2000) for 2 h at 4 °C. Protein bands were detected using Immobilon Western Chemiluminescent HRP Substrate (Millipore, Darmstadt, Germany; Cat. No. WBKLS0500) and imaged with an e-BLOT Touch Imaging System (e-BLOT, Shanghai, China). All experiments were performed with three technical replicates and validated across three independent biological repeats to ensure reproducibility.

### Cell counting kit‑8 (CCK‑8) assay

For the CCK-8 assay, lung cancer cells were seeded into 96-well plates at a density of 5,000 cells/well in complete medium and allowed to adhere overnight. At the indicated time points (0, 24, 48, and 72 h), 10 µL of CCK-8 reagent (Dojindo, Kumamoto, Japan; Cat. No. CK04) was added to each well. After a 2 h incubation at 37 °C in 5% CO_2_, absorbance at 450 nm was measured using a Multiskan™ FC Microplate Photometer (Thermo Fisher Scientific; Cat. No. 51119080). Cell viability was normalized to the 0 h control group. All assays were performed with three technical replicates and validated across three independent biological repeats to ensure statistical rigor and reproducibility.

### Scratch assay

Lung cancer cells were seeded at a density of 5 × 10^5^ cells per well in 6-well plates and cultured to 90–95% confluence. A standardized wound was generated in the monolayer using a 200-µL sterile pipette tip, followed by three washes with PBS to remove detached cells. Cells were then maintained in serum-free RPMI-1640 medium (Gibco). Wound images were captured at 0 and 24 h using a Nikon ECLIPSE Ts2-FL inverted microscope (20× objective; Nikon, Tokyo, Japan) equipped with a DS-Qi2 digital camera. Wound width was measured at three predefined positions per well using ImageJ software (version 1.54f; NIH, Bethesda, MD, USA) with the Wound Healing Size Tool plugin. Relative wound closure (%) was calculated as: wound closure = 100 × (Width_0 h_ − Width_24 h_)/Width_0 h_. Each experiment was performed with three technical replicates and three independent biological repeats.

### Transwell assay

Cell migration and invasion assays were performed using 8-µm pore Transwell chambers (Labselect, Beijing, China; Cat. No. 14341). For invasion assays, chambers were pre-coated with Matrigel matrix (Corning; Cat. No. 356234). Matrigel was thawed overnight at 4 °C, diluted 1:8 in ice-cold serum-free RPMI-1640 medium (Gibco), and 60 µL of the diluted solution was added to the upper chamber on ice. After incubation at 37 °C for 3 h to allow polymerization, excess Matrigel was removed by rinsing with serum-free RPMI-1640. HCC827 or H1975 cells (5 × 10^4^) were suspended in 100 µL serum-free RPMI-1640 and seeded into the upper chamber, while 500 µL RPMI-1640 containing 10% FBS was added to the lower chamber as a chemoattractant. After 15 h of incubation at 37 °C in 5% CO_2_, non-migrated cells on the upper membrane surface were gently removed with a cotton swab. Migrated or invaded cells were fixed with 4% paraformaldehyde (PFA) for 30 min, stained with 0.1% crystal violet for 15 min, and washed three times with PBS. Images were captured at 100× magnification using a Nikon ECLIPSE Ts2-FL inverted microscope equipped with a DS-Qi2 digital camera (Nikon, Tokyo, Japan). Five random fields per membrane were analyzed, and cell counts were obtained using ImageJ software (version 1.54f; NIH). Migration assays were performed under identical conditions but without Matrigel coating. Each experiment included three technical replicates and three independent biological repeats.

### Flow cytometry assay

Lung cancer cells (1 × 10^6^ cells/mL) were washed three times with PBS and resuspended in 200 µL binding buffer. Cells were stained using the Annexin V-FITC/PI Apoptosis Detection Kit (MultiSciences, Hangzhou, China; Cat. No. AP101) by adding 5 µL Annexin V-FITC and 5 µL propidium iodide (PI) per 100 µL cell suspension, followed by incubation in the dark for 30 min at room temperature. Apoptosis was analyzed immediately using a CytoFLEX S flow cytometer (Beckman Coulter, Brea, CA, USA) equipped with a 488-nm laser and 525/40 nm (FITC) and 690/50 nm (PI) filters. Cell populations were classified as viable (Annexin V^−^/PI^−^), early apoptotic (Annexin V^+^/PI^−^), late apoptotic (Annexin V^+^/PI^+^), or necrotic (Annexin V^−^/PI^+^). Data were analyzed using FlowJo software (version 10.3; BD Life Sciences, Ashland, OR, USA), with 10,000 events collected per sample. All assays were performed in triplicate and validated across three independent biological repeats to ensure statistical rigor and reproducibility.

### Xenograft tumor model

Six-week-old male BALB/c nude mice (20–25 g) were obtained from Hangzhou Medical College (Hangzhou, China; Certificate No. SCXK(Zhe)2024–0037). Mice were maintained under specific pathogen-free (SPF) conditions at the Animal Laboratory of The First Affiliated Hospital, Zhejiang University School of Medicine. All in vivo experiments were approved by, and conducted in accordance with, the Institutional Animal Care and Use Committee (IACUC) of The First Affiliated Hospital, Zhejiang University School of Medicine (Protocol No. (2025) SYDW-No. 139). Stable transfected HCC827 and H1975 cell lines were used to establish xenograft models. Each mouse received a subcutaneous injection of 2 × 10^6^ cells suspended in 100 µL PBS into the right flank. When tumors became palpable (approximately 50 mm^3^), mice were randomly assigned to experimental groups (*n* = 6 per group; see Table 1) using a random number table to ensure comparable baseline tumor burdens. Tumor measurements and endpoint analyses were performed by investigators blinded to group allocation. Tumor growth was monitored weekly by measuring length (L) and width (W) with digital calipers. Tumor volume (mm^3^) was calculated as V = 0.5 × L × W^2^. Mice were euthanized when tumor length reached 15 mm, at which point xenografts were excised for further analyses. Euthanasia was performed by CO_2_ inhalation followed by cervical dislocation, in accordance with institutional animal care guidelines.

**Table 1:**
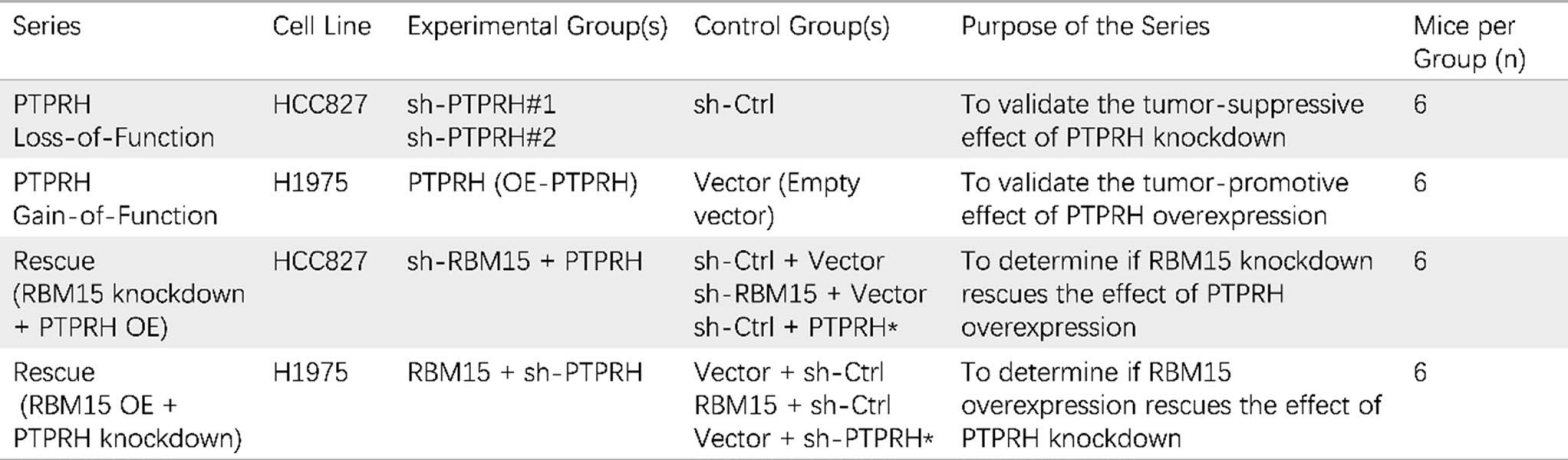
Experimental design and group allocation for the xenograft tumor model

### Immunohistochemistry (IHC)

Immunohistochemistry was performed on formalin-fixed, paraffin-embedded (FFPE) sections of human lung cancer specimens and mouse xenograft tumor tissues. The staining service was provided by Hangzhou Haokebio (Hangzhou, China). For human specimens, sections were incubated with primary antibodies against PTPRH (rabbit, Abmart, Shanghai, China; Cat. No. PK98451; 1:200), IGF2BP2 (rabbit, Proteintech; Cat. No. 11601–1-AP; 1:200), and Ki67 (rabbit, Haoke, Hangzhou, China; Cat. No. B11002R; 1:500). For xenograft tissues, apoptosis was assessed by TUNEL staining (Haoke; Cat. No. HKI0012), and proliferation was evaluated with Ki67 (rabbit, Haoke; Cat. No. B11002R; 1:500). Briefly, after deparaffinization and antigen retrieval, sections were incubated with the primary antibodies overnight at 4 °C. The following day, sections were treated with the appropriate secondary antibody and visualized with a diaminobenzidine (DAB) substrate, followed by hematoxylin counterstaining.

For image acquisition and quantitative analysis, all stained slides were scanned and digitized using an EVOS M7000 whole-slide scanner (Thermo Fisher Scientific) equipped with a 20× objective lens. Five representative fields per sample were selected for image capture. Staining was independently evaluated by two experienced investigators blinded to the clinical data.

For scoring system and statistical analysis, protein expression levels (e.g., PTPRH, RBM15, and IGF2BP2) were semi-quantitatively assessed using the H-score method. The H-score was calculated as the product of staining intensity (0, negative; 1, weak; 2, moderate; 3, strong) and the percentage of positive tumor cells (0–100%), yielding a continuous score ranging from 0 to 300. All samples were included in the analysis as continuous variables. For statistical comparisons, the mean H-scores of tumor tissues were compared with those of adjacent normal tissues using a paired Student’s *t*-test or Wilcoxon signed-rank test, as appropriate, without applying arbitrary dichotomization. For Ki67 staining in xenograft tissues, the proliferation index was defined as the percentage of positively stained tumor nuclei relative to the total number of tumor cells across five representative fields. Quantitative analysis of staining intensity and positive area was performed using ImageJ software (version 1.54f; NIH).

### Methylated RNA immunoprecipitation sequencing (MeRIP-seq)

Total RNA was extracted, purified, and fragmented into ~100-nucleotide fragments using the Magna MeRIP m6A Kit (Cat. No. 17–10499; Millipore, Billerica, MA, USA). m6A-modified RNA fragments were enriched using immunomagnetic beads conjugated with an anti-m6A antibody. The RNA–protein complexes were subsequently isolated, and the enriched RNA was reverse-transcribed into cDNA for library construction. High-throughput sequencing was performed on an Illumina NovaSeq™ 6000 platform (Illumina, San Diego, CA, USA) using the paired-end 150 bp (PE150) mode. Sequencing and downstream bioinformatics analyses were conducted by Hangzhou Kaitai Biotechnology Co., Ltd. (Hangzhou, China). To facilitate independent validation of the key findings related to PTPRH, the complete set of differential m6A peak data for this gene, including genomic coordinates, statistical significance, and enrichment scores, is provided in Supplementary Table S4.

### m6A-RNA immunoprecipitation–qPCR (MeRIP-qPCR)

The m6A immunoprecipitation (m6A-IP) assay was performed using the Magna RIP™ RNA-Binding Protein Immunoprecipitation Kit (Millipore; Cat. No. 17–700) according to the manufacturer’s protocol. Briefly, magnetic protein A/G beads were incubated with 5 µg of anti-m6A mouse monoclonal antibody (Proteintech; Cat. No. 68055–1-Ig) or normal mouse IgG (negative control) in RIP Immunoprecipitation Buffer for 30 min at room temperature with gentle rotation to generate antibody–bead complexes. Approximately 2 × 10^7^ lung cancer cells per sample were harvested and lysed in complete RIP Lysis Buffer. Lysates were centrifuged at 14,000 rpm for 10 min at 4 °C, and the supernatant was collected. A portion of the supernatant was saved as the “Input” control, while the remaining lysate was equally divided and incubated overnight at 4 °C with the antibody–bead complexes under gentle rotation. The next day, bead complexes were extensively washed with RIP Wash Buffer, and bound m6A-modified RNAs were eluted by Proteinase K digestion followed by RNA purification. Co-precipitated RNA from the m6A-IP, IgG, and Input groups was reverse-transcribed into cDNA, and enrichment of *PTPRH* mRNA was quantified by RT-qPCR. Relative m6A enrichment was calculated by normalizing the m6A-IP signal to the Input control after subtraction of background signal from the IgG control. Fold enrichment was determined using the comparative ΔΔCt method as follows:

ΔCt _normalized IP_ = [Average Ct _IP_- Average Ct _input_- log_2_(input dilution factor)]

ΔCt _normalized IgG_ = [Average Ct _IgG_- Average Ct _input_- log_2_(input dilution factor)]

ΔΔCt = ΔCt _normalized IP_- ΔCt _normalized IgG_

Fold enrichment = 2^-ΔΔCt^

### RNA immunoprecipitation (RIP)

RNA immunoprecipitation was performed using the RIP Kit (Gisece Biotech, Guangzhou, China; Cat. No. P0101) according to the manufacturer’s instructions. Briefly, approximately 1 × 10^7^ lung cancer cells were harvested and lysed in complete RIP lysis buffer. After centrifugation at 4 °C, the clarified supernatant was incubated overnight at 4 °C with magnetic beads conjugated to either anti-RBM15 or anti-IGF2BP2 antibodies, with normal IgG used as a negative control. The bead–antibody complexes were washed extensively with RIP Wash Buffer, and bound RNA–protein complexes were eluted. To eliminate genomic DNA contamination, the eluates were treated with DNase using the purification columns provided in the kit. RNA was purified according to the manufacturer’s protocol and analyzed by RT-qPCR to assess the fold enrichment of *PTPRH* mRNA.

### RNA pull-down assay and mass spectrometry

An RNA pull-down assay coupled with liquid chromatography–tandem mass spectrometry (LC-MS/MS) was performed by Shanghai GenePharma (Shanghai, China) to identify proteins interacting with PTPRH mRNA. Briefly, in vitro–transcribed, biotin-labeled PTPRH RNA (both sense and antisense transcripts) was incubated with whole-cell lysates prepared from H1975 cells stably overexpressing PTPRH. RNA–protein complexes were captured using streptavidin-coated magnetic beads, followed by extensive washing to remove nonspecifically bound proteins. Bound proteins were subsequently eluted and subjected to LC–MS/MS analysis for protein identification.

LC–MS/MS analysis was carried out using a proteomics platform provided by BGI Genomics Co., Ltd. (Shenzhen, China). Peptide mixtures were separated by nano-flow liquid chromatography on a Thermo Ultimate 3000 ultra-high-performance liquid chromatography (UHPLC) system (Thermo Fisher Scientific) and analyzed by tandem mass spectrometry using an Orbitrap Exploris 480 instrument (Thermo Fisher Scientific) operated in data-dependent acquisition (DDA) mode. Raw MS/MS data were processed using BGI’s proprietary bioinformatics pipeline and searched against the UniProtKB/Swiss-Prot human database. The search parameters were set as follows: trypsin was specified as the protease, allowing up to two missed cleavages; precursor and fragment mass tolerances were set to 20 ppm; carbamidomethylation of cysteine residues (C) was defined as a fixed modification; and oxidation of methionine (M), N-terminal glutamine to pyroglutamate conversion (Gln→pyro-Glu), and deamidation of asparagine and glutamine (NQ) were included as variable modifications. The false discovery rate (FDR) was controlled at ≤1% at both the peptide-spectrum match (PSM) and protein levels.

### Dual luciferase report assay

Dual-luciferase reporter plasmids were commercially constructed by Shanghai GenePharma Co., Ltd. (Shanghai, China). Briefly, the 3′-UTR fragment of human PTPRH was cloned downstream of the Renilla luciferase gene in the GP-miRGLO vector. Two constructs were generated, containing either the wild-type sequence or a mutant m6A motif. An empty GP-miRGLO vector without insert was used as a baseline control. Functional assays were performed in H1975 cells with stable knockdown or overexpression of RBM15 or IGF2BP2, as confirmed by Western blot analysis. Cells were seeded at a density of 5 × 10^4^ cells per well in 24-well plates 24 h prior to transfection. Upon reaching 80–90% confluence, cells were transfected with 400 ng of reporter plasmid (Vector, WT, or Mut) using Lipofectamine 3000 reagent (Cat. No. L3000001; Invitrogen) according to the manufacturer’s instructions. At 48 h post-transfection, luciferase activities were measured using the Dual-Luciferase® Reporter Assay System (Cat. No. E1910; Promega, Madison, WI, USA). Renilla luciferase activity was normalized to Firefly luciferase activity for each well (Renilla/Firefly). Data are presented as mean ± standard deviation (SD) from three independent experiments.

### Tube formation assay

Conditioned medium was collected from lung cancer cell cultures and used for HUVEC assays. HUVECs were seeded at a density of 1 × 10^5^ cells per well in Matrigel-coated 24-well plates (Corning) and initially cultured in serum-free medium for 12 h. For VEGF-neutralization experiments, conditioned medium was pre-incubated with 1 µg/mL bevacizumab (Cat. No. HY-P9906; MedChemExpress) or an equivalent concentration of human IgG1 kappa isotype control (Cat. No. HY-P99001; MedChemExpress) for 1 h at 37 °C. Cells were then incubated with the treated conditioned medium for an additional 6 h. Tube formation was evaluated by quantifying the number of nodes and the total tube length (pixels) per field of view using ImageJ software (version 1.54f; NIH). Each experiment was performed with three technical replicates and three independent biological repeats.

### VEGF determination by ELISA

Secreted VEGF levels were quantified using a Human VEGF Quantikine ELISA Kit (Cat. No. DVE00; R&D Systems, Minneapolis, MN, USA) according to the manufacturer’s instructions. Conditioned medium was collected from lung cancer cell cultures and subjected to ELISA analysis. Absorbance at 450 nm was measured using a Multiskan™ FC Microplate Photometer (Cat. No. 51119080; Thermo Fisher Scientific) following the addition of stop solution. Each experiment was performed with three technical replicates and three independent biological repeats.

### RNA stability assay

NSCLC cells were seeded in 6-well plates at 5 × 10^5^ cells per well and cultured to approximately 80% confluence. Cells were then treated with Actinomycin D (MedChemExpress; Cat. No. HY-17559) at 5 µg/mL for 0, 2, 4, 6, or 8 h. Total RNA was extracted using TRIzol reagent (Invitrogen), and target RNA levels were quantified by RT-qPCR. RNA isolation and RT-qPCR procedures were performed as described above. Each experiment was conducted with three technical replicates and three independent biological repeats.

### Statistical analyses

All statistical analyses were performed using GraphPad Prism (version 9.5.0; GraphPad Software, San Diego, CA, USA). Data from at least three independent biological replicates are presented as mean ± SD. Comparisons between matched clinical specimens were conducted using paired Student’s t-tests, whereas comparisons between two independent groups of cell samples were performed using unpaired Student’s *t*-tests. For comparisons involving multiple groups, one-way or two-way analysis of variance (ANOVA) was applied as appropriate, followed by Tukey’s post hoc test to evaluate differences among groups or the effects of two independent variables (e.g., treatment and time). Survival curves were generated using the Kaplan–Meier method and compared using the log-rank test. A two-sided *p* value < 0.05 was considered statistically significant.

## Results

### PTPRH is overexpressed in NSCLC and associated with poor prognosis

**Fig. 1:**
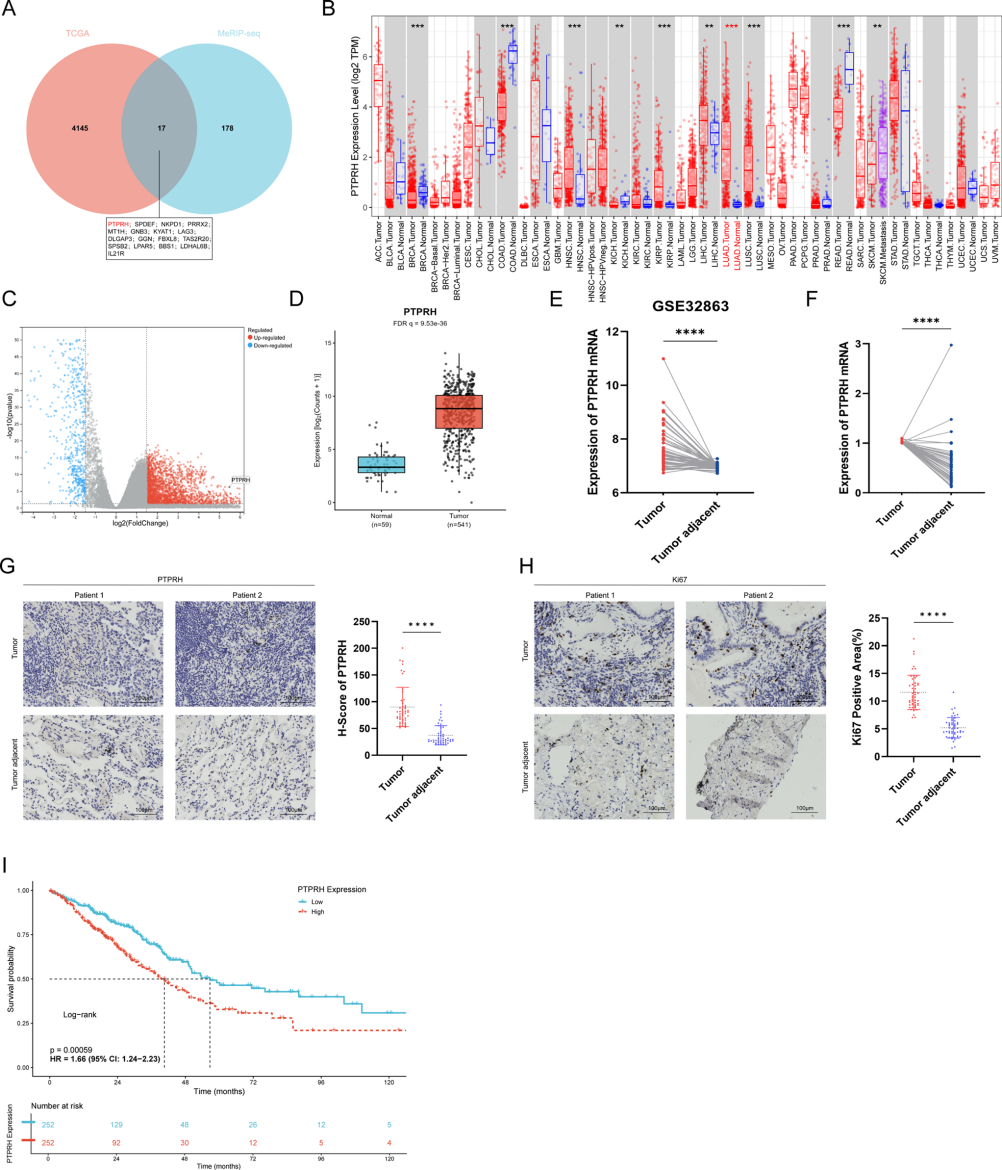
*PTPRH* is overexpressed in lung cancer and associated with poor prognosis. (**A**) Venn diagram illustrating the overlap between genes with elevated m6A methylation (MeRIP-seq) and significantly upregulated genes in the TCGA-LUAD cohort. (**B**) Comparative analysis of *PTPRH* expression between tumor and normal tissues in TCGA. (**C**) Volcano plot showing differential *PTPRH* expression in TCGA-LUAD. (**D**) *PTPRH* expression based on TCGA transcriptomic data (Normal = 59, Tumor = 541; log2FC = 5.16, 95% CI: 3.48–6.83, FDR q = 9.53 × 10^−36^). (**E**) PTPRH mRNA expression in paired tumor and adjacent normal tissues from the GSE32863 dataset (*n* = 58; paired *t*-test; fold change [Tumor/Normal] = 1.10, *p* < 0.0001). (**F**) qPCR analysis of *PTPRH* in paired tumor and normal tissues (*n* = 50), with three technical replicates per sample (paired two-tailed *t*-test; fold change [Tumor/Normal] = 1.57, *p* < 0.0001). (**G, H**) Representative IHC staining of PTPRH and Ki67 in LUAD and adjacent tissues (scale bar = 100 μm), with quantification from 50 paired samples based on five randomly selected fields per sample (paired *t*-test; PTPRH fold change = 2.42, *p* < 0.0001; Ki67 fold change = 2.23, *p* < 0.0001). (**I**) Kaplan–Meier analysis of overall survival (OS) stratified by *PTPRH* expression (n_high = 252, n_low = 252), with the cutoff defined by median expression (log-rank test; HR = 1.66, 95% CI: 1.24–2.23, *p* = 0.00059). The number of patients at risk is shown below the plot

To identify potential m6A-modified oncogenes, we integrated epigenomic and transcriptomic datasets. In a previous study [16], we showed that RBM15 overexpression increases global m6A methylation and identified 195 genes with significantly hypermethylated peaks (log_2_|FC| >1, *p* < 0.05). Concurrently, analysis of the TCGA-LUAD cohort revealed 4,162 genes upregulated in tumor tissues relative to normal controls (log_2_|FC| >1, *p* < 0.05). The intersection of these datasets identified 17 genes with both elevated m6A methylation and increased expression in LUAD (Fig. 1A). Among these, PTPRH was prioritized for further investigation given its established role in tyrosine phosphatase signaling and its reported oncogenic functions.

Comprehensive TCGA analysis demonstrated aberrant PTPRH expression across multiple cancer types, with particularly high levels in LUAD (Fig. 1B). Survival analyses revealed significant disparities between patients with high versus low PTPRH expression across independent cohorts. In the TCGA-LUAD cohort specifically, elevated PTPRH was associated with significantly shorter overall survival (OS) (Supplementary Fig. S1). A volcano plot of TCGA-LUAD data positioned PTPRH among the most upregulated genes (log_2_|FC| = 5.485, *p* < 0.001) (Fig. 1C). Re-analysis of TCGA RNA-seq data further confirmed that *PTPRH* expression was significantly higher in tumor tissues than in normal tissues (Fig. 1D). Moreover, multivariable Cox regression analysis supported the independent prognostic value of PTPRH (Table 2).

Independent validation using the GSE32863 dataset also demonstrated significantly elevated PTPRH expression in lung cancer tissues relative to adjacent normal tissues (Fig. 1E). Consistent with these findings, qPCR and immunohistochemistry performed on 50 paired clinical samples confirmed that PTPRH mRNA and protein levels were markedly upregulated in tumors (Fig. 1F, G). Elevated PTPRH expression correlated with increased Ki67 staining in the same specimens, supporting its association with enhanced proliferation (Fig. 1H). Furthermore, clinicopathological analysis revealed a significant association between high PTPRH protein expression and lymph node metastasis (N stage) (Supplementary Table 2). Kaplan-Meier analysis further confirmed that high PTPRH expression was significantly associated with worse OS (Fig. 1I). Collectively, these findings strongly implicate PTPRH as an important driver of lung cancer pathogenesis and progression.

**Table 2.**
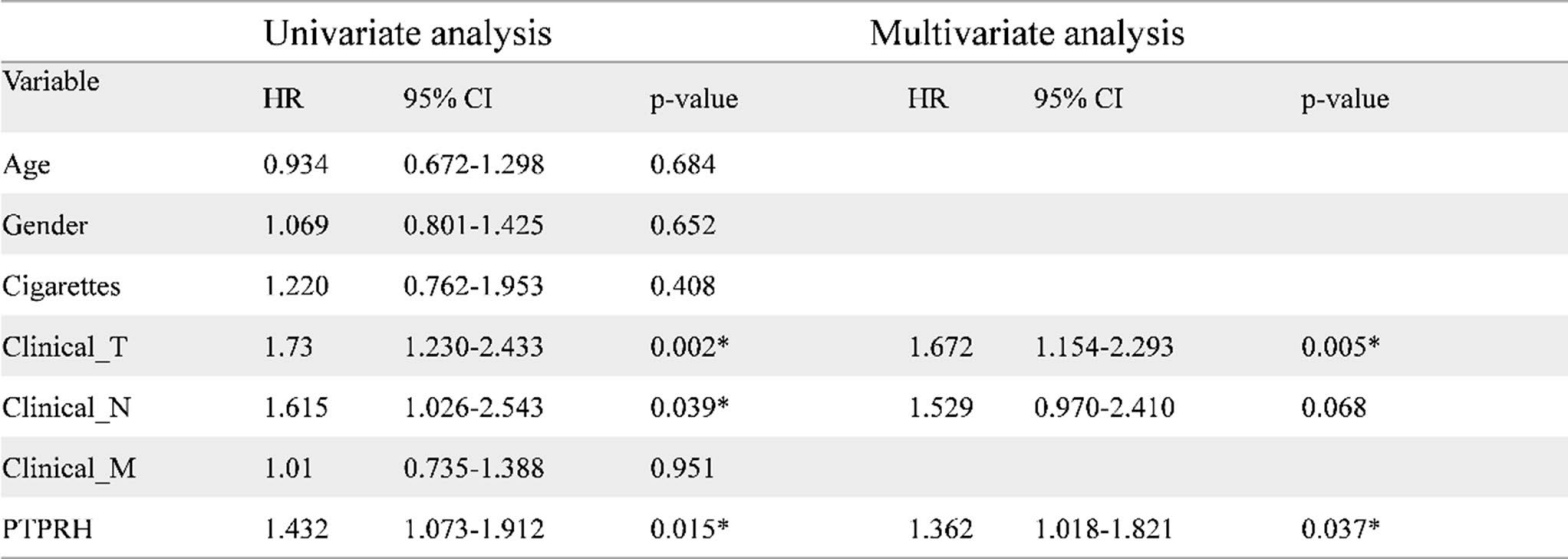
Univariate and multivariate analyses of the factors correlated with the overall survival of LUAD patients. (*Statistically significant, *p* < 0.05)

### PTPRH promotes the proliferation, migration, and invasion of NSCLC cells in vitro and in vivo

**Fig. 2:**
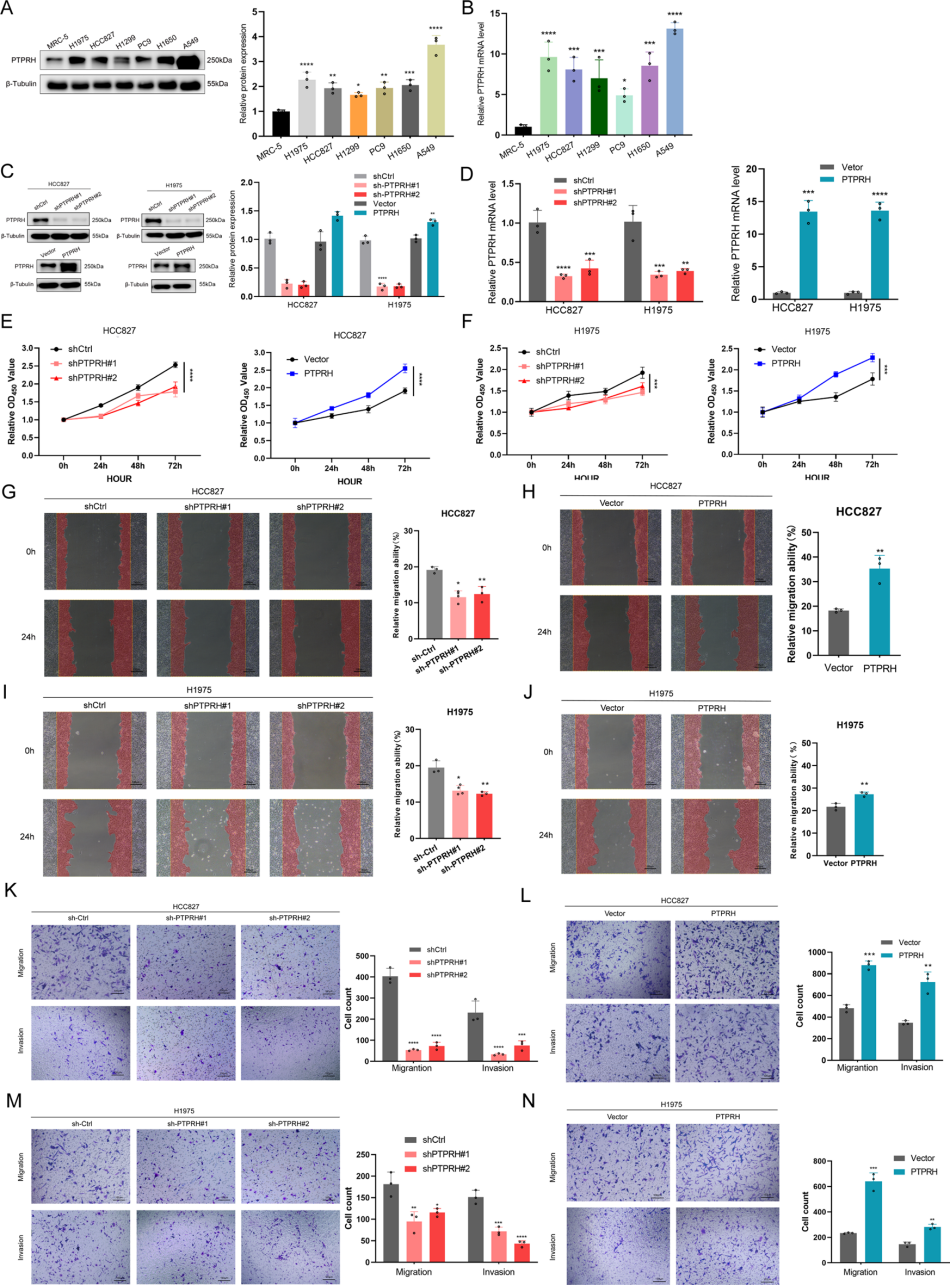
PTPRH promotes proliferation, migration, and invasion in vitro. (**A**) PTPRH expression in lung cancer cell lines detected by Western blot (*n* = 3 independent experiments, normalized to β-Tubulin). One-way ANOVA with Dunnett’s test was performed. Fold changes relative to MRC-5 were as follows: H1975 = 2.27 (*p* < 0.0001), HCC827 = 1.93 (*p* = 0.0013), H1299 = 1.62 (*p* = 0.019), PC9 = 1.94 (*p* = 0.0012), H1650 = 2.05 (*p* = 0.0005), and A549 = 3.68 (*p* < 0.0001). (**B**) *PTPRH* expression in lung cancer cell lines measured by qPCR (*n* = 3 independent experiments). One-way ANOVA with Dunnett’s test was performed. Fold changes relative to MRC-5 were as follows: H1975 = 9.63 (*p* < 0.0001), HCC827 = 8.09 (*p* = 0.0002), H1299 = 7.02 (*p* = 0.0009), PC9 = 4.89 (*p* = 0.0269), H1650 = 8.54 (*p* = 0.0001), and A549 = 13.12 (*p* < 0.0001). (**C**) Western blot validation of PTPRH knockdown and overexpression (*n* = 3 independent experiments, normalized to β-Tubulin). Representative blots are shown. In the upper panel, knockdown effects in HCC827 and H1975 cells were analyzed by one-way ANOVA with Dunnett’s test, showing fold changes relative to sh-Ctrl as follows: HCC827-shPTPRH#1 = 0.23 (*p* < 0.0001), shPTPRH#2 = 0.20 (*p* < 0.001); H1975-shPTPRH#1 = 0.18 (*p* < 0.0001), shPTPRH#2 = 0.19 (*p* < 0.0001). In the lower panel, overexpression effects were analyzed using an unpaired *t*-test relative to Vector, showing fold changes of 1.47 in HCC827 (*p* = 0.0141) and 1.31 in H1975 (*p* = 0.0034). (**D**) RT-qPCR validation of PTPRH knockdown and overexpression (*n* = 3 independent experiments). Knockdown effects in HCC827 and H1975 cells were analyzed by one-way ANOVA with Dunnett’s test, showing fold changes relative to sh-Ctrl as follows: HCC827-shPTPRH#1 = 0.33 (*p* < 0.0001), shPTPRH#2 = 0.42 (*p* = 0.0002); H1975-shPTPRH#1 = 0.34 (*p* < 0.0001), shPTPRH#2 = 0.39 (*p* = 0.0001). Overexpression effects were analyzed using an unpaired *t*-test relative to Vector, showing fold changes of 13.5 in HCC827 (*p* = 0.0002) and 13.6 in H1975 (*p* < 0.0001). (**E, F**) Cell proliferation assessed by CCK-8 assay (*n* = 3 independent experiments). In HCC827 cells (**E**), knockdown effects were analyzed by two-way ANOVA with Dunnett’s test (interaction: F(6,45) = 42.72, *p* < 0.0001), showing fold changes at 72 h of shPTPRH#1 = 0.70 (*p* < 0.0001) and shPTPRH#2 = 0.76 (*p* = 0.0001), while overexpression effects were analyzed by two-way ANOVA with Sidak’s test (interaction: F(3,30) = 29.21, *p* < 0.0001), with a fold change of 1.33 (*p* < 0.0001). In H1975 cells (**F**), knockdown effects were analyzed by two-way ANOVA with Dunnett’s test (interaction: F(6,45) = 10.89, *p* < 0.0001), showing fold changes at 72 h of shPTPRH#1 = 0.76 (*p* = 0.0008) and shPTPRH#2 = 0.84 (*p* = 0.0103), whereas overexpression effects were analyzed by two-way ANOVA with Sidak’s test (interaction: F(3,30) = 21.94, *p* < 0.0001), with a fold change of 1.28 (*p* = 0.0003). (**G**–**J**) Cell migration evaluated by scratch assay (scale bar = 100 μm, *n* = 3 independent experiments). In HCC827 cells (**G, H**), knockdown reduced wound closure to 60.4% (*p* = 0.003) and 64.8% (*p* = 0.005) relative to sh-Ctrl (one-way ANOVA with Dunnett’s test), whereas overexpression increased wound closure to 193.4% compared with Vector (*p* = 0.006, unpaired *t*-test). In H1975 cells (**I, J**), knockdown reduced wound closure to 67.4% (*p* = 0.003) and 63.0% (*p* = 0.001) relative to sh-Ctrl (one-way ANOVA with Dunnett’s test), whereas overexpression increased wound closure to 125.8% compared with Vector (*p* = 0.006, unpaired *t*-test). (**K**–**N**) Cell migration and invasion assessed by Transwell assays. In HCC827 cells (**K, L**), knockdown significantly reduced migration (shPTPRH#1 = 0.13, *p* < 0.0001; shPTPRH#2 = 0.18, *p* < 0.0001) and invasion (shPTPRH#1 = 0.14, *p* = 0.0007; shPTPRH#2 = 0.33, *p* = 0.0026) relative to sh-Ctrl (one-way ANOVA with Dunnett’s test), whereas overexpression increased migration (fold change = 1.83, *p* = 0.0002) and invasion (fold change = 2.08, *p* = 0.0025) (unpaired *t*-test). In H1975 cells (**M, N**), knockdown reduced migration (shPTPRH#1 = 0.52, *p* = 0.0048; shPTPRH#2 = 0.64, *p* = 0.0174) and invasion (shPTPRH#1 = 0.48, *p* = 0.0003; shPTPRH#2 = 0.29, *p* < 0.0001), whereas overexpression increased migration (fold change = 2.74, *p* = 0.0005) and invasion (fold change = 1.93, *p* = 0.0012)

To further elucidate the functional role of PTPRH, we first examined its expression across a panel of lung cancer cell lines in comparison with the human embryonic lung fibroblast cell line MRC-5. PTPRH expression was markedly elevated in all cancer cell lines at both the mRNA and protein levels (Fig. 2A, B). Based on these results, HCC827 and H1975 cells were selected for subsequent analyses. Stable knockdown constructs (sh-PTPRH) and an overexpression plasmid (OE-PTPRH), along with their respective control vectors, were established and validated by Western blot analysis and qPCR (Fig. 2C, D). Functional assays demonstrated that PTPRH knockdown significantly inhibited cell proliferation in both cell lines, whereas overexpression promoted proliferation (Fig. 2E, F). Wound-healing assays revealed that silencing of PTPRH impaired migratory capacity, while its overexpression enhanced cell migration (Fig. 2G–J). Consistently, Transwell assays showed that PTPRH knockdown significantly reduced invasive ability, whereas overexpression markedly increased invasion (Fig. 2K–N). These pro-tumorigenic effects of PTPRH were further validated in KRAS-mutant LUAD (A549) and LUSC (H520) cell lines, as shown in Supplementary Fig. 2.

**Fig. 3:**
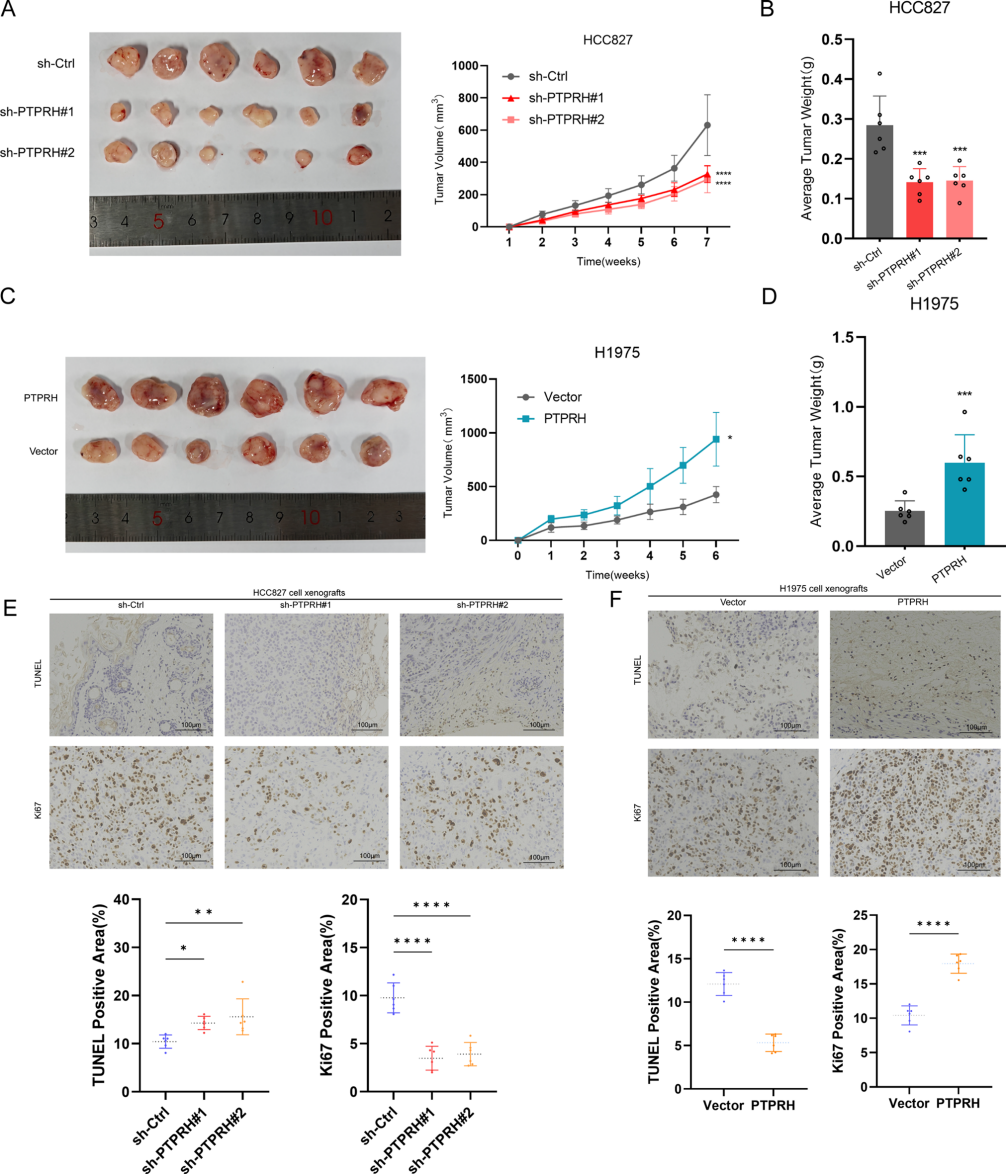
PTPRH regulates tumor growth and apoptosis in vivo. (**A**, **B**) Effects of PTPRH knockdown on tumor growth in HCC827 xenograft models (*n* = 6 mice per group). (**A**) Tumor volume was measured weekly over 7 weeks and analyzed by two-way ANOVA with Dunnett’s test. Fold changes at week 7 relative to sh-Ctrl were 0.52 for sh-PTPRH#1 (*p* < 0.0001) and 0.47 for sh-PTPRH#2 (*p* < 0.0001). (**B**) Final tumor weights at necropsy were analyzed by one-way ANOVA with Dunnett’s test, showing fold changes of 0.49 for sh-PTPRH#1 (*p* = 0.0005) and 0.51 for sh-PTPRH#2 (*p* = 0.0007) relative to sh-Ctrl. (**C, D**) Effects of PTPRH overexpression on tumor growth in H1975 xenograft models (*n* = 6 mice per group). (**C**) Tumor volume was measured weekly over 6 weeks and analyzed by two-way ANOVA with Sidak’s test, showing a fold change of 2.21 at week 6 compared with Vector (*p* < 0.0001). (**D**) Final tumor weights at necropsy were analyzed using an unpaired *t*-test, showing a fold change of 2.38 relative to Vector (*p* = 0.0026). (**E, F**) Representative immunohistochemical images of TUNEL and Ki67 staining in xenograft tumors (scale bar = 100 μm, *n* = 6 mice per group). In HCC827 xenografts (**E**) PTPRH knockdown increased apoptosis, as indicated by TUNEL staining (one-way ANOVA with Dunnett’s test; positive area: sh-PTPRH#1 = 1.37, *p* = 0.0273; sh-PTPRH#2 = 1.50, *p* = 0.0043), and reduced proliferation, as indicated by Ki67 staining (positive area: sh-PTPRH#1 = 0.36, *p* < 0.0001; sh-PTPRH#2 = 0.40, *p* < 0.0001). In H1975 xenografts (**F**) PTPRH overexpression decreased apoptosis (TUNEL staining; fold change = 0.44, *p* < 0.0001) and increased proliferation (Ki67 staining; fold change = 1.72, *p* < 0.0001), as determined by unpaired *t*-test relative to Vector

The oncogenic role of PTPRH was further validated in vivo using subcutaneous xenograft models in six-week-old male nude mice. Tumors derived from HCC827 cells with PTPRH knockdown exhibited significantly reduced volume and weight compared with controls (Fig. 3A, B), whereas tumors derived from H1975 cells overexpressing PTPRH showed significant increases in both parameters (Fig. 3C, D). To examine proliferation and apoptosis in vivo, xenograft tissues were subjected to Ki67 and TUNEL staining. Tumors with PTPRH knockdown displayed reduced Ki67 positivity and increased TUNEL staining, indicating suppressed proliferation and enhanced apoptosis (Fig. 3E). Conversely, PTPRH-overexpressing tumors showed increased Ki67 staining and reduced TUNEL positivity, consistent with enhanced proliferation and reduced apoptosis (Fig. 3F).

### PTPRH modulates apoptosis and pro-angiogenic signaling consistent with VEGF/HIF-1α pathway activation

**Fig. 4:**
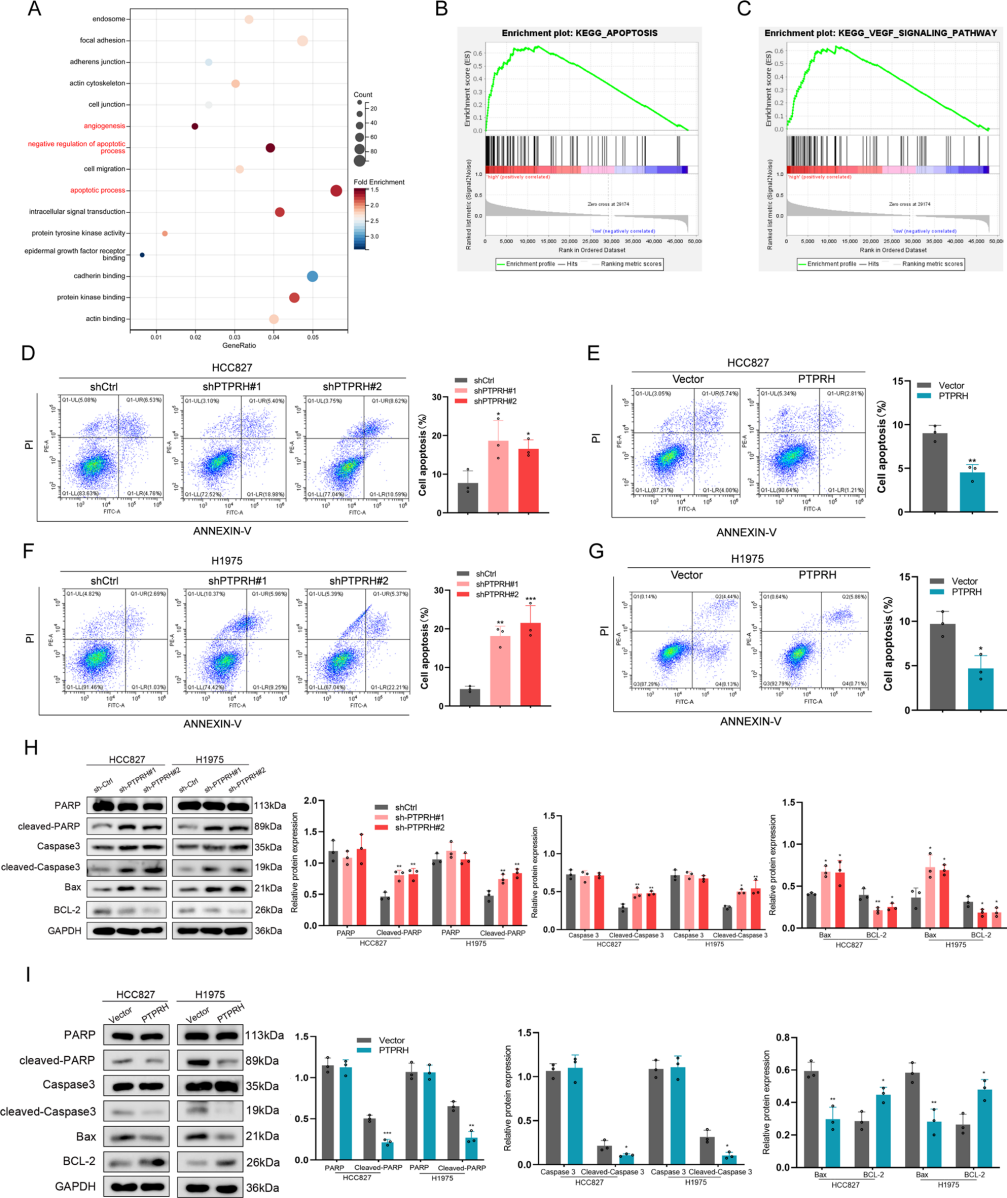
PTPRH promotes tumor progression by regulating apoptosis. (**A**) Gene Ontology enrichment analysis of genes positively correlated with *PTPRH*. (**B**, **C**) Gene Set Enrichment Analysis (GSEA) demonstrating significant associations with apoptosis and VEGF signaling pathways. (**D**–**G**) Apoptosis assessed by flow cytometry. In HCC827 cells (**D**, **E**), knockdown of PTPRH increased apoptosis (one-way ANOVA with Dunnett’s test; fold change: shPTPRH#1 = 2.41, *p* = 0.0209; shPTPRH#2 = 2.14, *p* = 0.049), whereas overexpression reduced apoptosis (unpaired *t*-test; fold change = 0.41, *p* = 0.0024). In H1975 cells (**F**, **G**), PTPRH knockdown markedly increased apoptosis (one-way ANOVA with Dunnett’s test; fold change: shPTPRH#1 = 4.10, *p* = 0.0025; shPTPRH#2 = 4.87, *p* = 0.0008), whereas overexpression suppressed apoptosis (unpaired *t*-test; fold change = 0.50, *p* = 0.0036). (**H**, **I**) Expression of apoptosis-related proteins following PTPRH knockdown or overexpression (*n* = 3 independent experiments, normalized to GAPDH), with representative blots shown. In knockdown experiments (**H**), one-way ANOVA with Dunnett’s test revealed no significant changes in total PARP or caspase-3 levels, but increased levels of cleaved PARP and cleaved caspase-3, along with upregulation of BAX and downregulation of BCL-2, in both HCC827 and H1975 cells. Specifically, in HCC827 cells, shPTPRH#1 and shPTPRH#2 increased cleaved PARP (1.73, *p* = 0.0035; 1.76, *p* = 0.0028) and cleaved caspase-3 (1.63, *p* = 0.008; 1.65, *p* = 0.0072), with corresponding increases in BAX (1.65, *p* = 0.0218; 1.62, *p* = 0.0258) and decreases in BCL-2 (0.53, *p* = 0.0092; 0.63, *p* = 0.0266). Similar trends were observed in H1975 cells, with increased cleaved PARP (1.56–1.76), cleaved caspase-3 (1.70–1.85), and BAX (1.89–1.98), and decreased BCL-2 (0.59–0.60). In overexpression experiments (**I**), unpaired *t*-test analysis showed reduced levels of cleaved PARP (HCC827: 0.43, *p* = 0.0006; H1975: 0.42, *p* = 0.0021) and cleaved caspase-3 (HCC827: 0.51, *p* = 0.045; H1975: 0.33, *p* = 0.0122), decreased BAX (HCC827: 0.50, *p* = 0.0051; H1975: 0.48, *p* = 0.0063), and increased BCL-2 (HCC827: 1.56, *p* = 0.0179; H1975: 1.81, *p* = 0.0136), while total PARP and caspase-3 levels remained unchanged

To investigate the pathways through which PTPRH exerts its oncogenic effects, genes positively correlated with PTPRH were extracted from the TCGA-LUAD dataset and subjected to Gene Ontology (GO) enrichment analysis. PTPRH was most strongly associated with angiogenesis- and apoptosis-related pathways (Fig. 4A). Consistently, single-gene Gene Set Enrichment Analysis (GSEA) demonstrated a close association with apoptosis and VEGF signaling (Fig. 4B, C). Functional validation supported these predictions. Flow cytometry showed that PTPRH knockdown significantly increased apoptosis in HCC827 and H1975 cells, whereas overexpression suppressed it (Fig. 4D–G). Western blot analysis further revealed that silencing PTPRH reduced BCL-2 expression while increasing BAX, cleaved caspase-3, and cleaved PARP, whereas overexpression produced the opposite pattern (Fig. 4H, I).

**Fig. 5:**
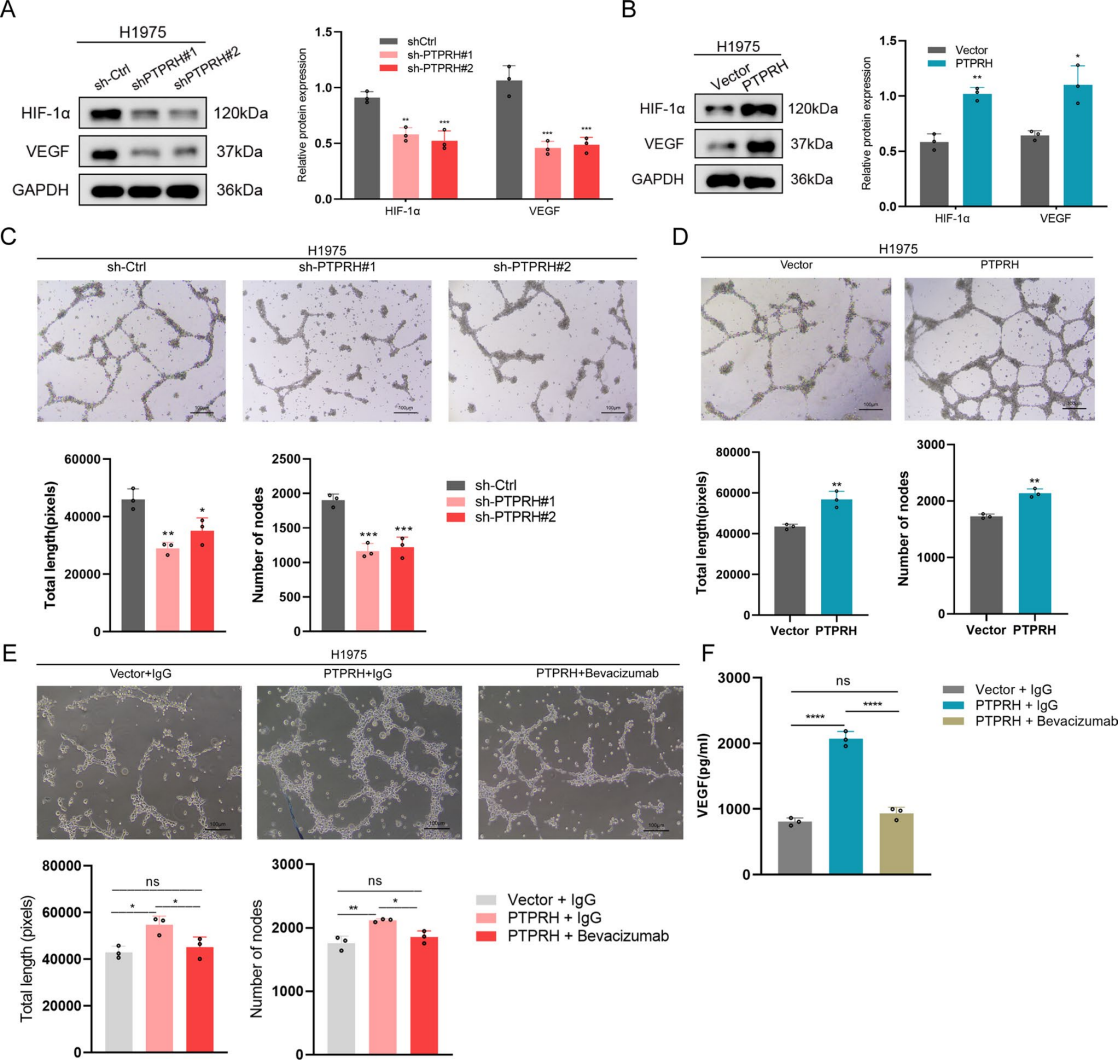
PTPRH promotes tumor progression by regulating VEGF signaling. (**A**, **B**) Expression of HIF-1α and VEGF following modulation of PTPRH (*n* = 3 independent experiments, normalized to GAPDH), with representative blots shown. (**A**) In H1975 cells, PTPRH knockdown reduced HIF-1α and VEGF expression (one-way ANOVA with Dunnett’s test; fold changes vs. sh-Ctrl: shPTPRH#1, HIF-1α = 0.63, *p* = 0.0019, VEGF = 0.43, *p* = 0.0004; shPTPRH#2, HIF-1α = 0.57, *p* = 0.0008, VEGF = 0.46, *p* = 0.0005). (**B**) PTPRH overexpression increased HIF-1α and VEGF levels (unpaired *t*-test; fold changes vs. Vector: HIF-1α = 1.74, *p* = 0.0012; VEGF = 1.71, *p* = 0.0112). (**C**, **D**) Tube formation assays assessing angiogenesis following PTPRH knockdown or overexpression (scale bar = 100 μm, *n* = 3 independent experiments). (**C**) Knockdown reduced angiogenic capacity, as indicated by decreased total tube length (shPTPRH#1 = 0.63, *p* = 0.0019; shPTPRH#2 = 0.76, *p* = 0.0161) and reduced number of nodes (shPTPRH#1 = 0.62, *p* = 0.0005; shPTPRH#2 = 0.64, *p* = 0.0007) relative to sh-Ctrl (one-way ANOVA with Dunnett’s test). (**D**) Overexpression enhanced angiogenesis, with increased total tube length (fold change = 1.31, *p* = 0.0053) and node number (fold change = 1.24, *p* = 0.0012) relative to Vector (unpaired *t*-test). (**E**) Tube formation assays evaluating the effect of bevacizumab (1 μg/mL) on PTPRH-induced angiogenesis (scale bar = 100 μm, *n* = 3 independent experiments; one-way ANOVA with Tukey’s test). PTPRH overexpression increased total tube length and node number in the presence of IgG control (fold changes vs. Vector + IgG: 1.27, *p* = 0.0192 and 1.20, *p* = 0.0049, respectively), whereas bevacizumab treatment significantly attenuated these effects (fold changes vs. PTPRH + IgG: 0.83, *p* = 0.0442 and 0.86, *p* = 0.0206, respectively). (**F**) ELISA analysis showing that bevacizumab (1 μg/mL) reduced the elevated levels of secreted VEGF induced by PTPRH overexpression (*n* = 3 independent experiments; one-way ANOVA with Tukey’s test; fold changes: PTPRH + IgG vs. Vector + IgG = 2.56, *p* < 0.0001; PTPRH + bevacizumab vs. PTPRH + IgG = 0.45, *p* < 0.0001)

Examination of VEGF pathway proteins showed that knockdown decreased HIF-1α and VEGF expression in H1975 cells, while overexpression enhanced their expression (Fig. 5A, B). In line with these molecular findings, tube formation assays demonstrated that PTPRH knockdown impaired vascular network formation, whereas overexpression significantly promoted angiogenesis (Fig. 5C, D). Notably, the pro-angiogenic effect induced by PTPRH overexpression was markedly attenuated by treatment with the VEGF-neutralizing antibody bevacizumab (Fig. 5E). In agreement with these findings, ELISA results showed that bevacizumab significantly reduced the elevated levels of secreted VEGF in conditioned medium derived from PTPRH-overexpressing cells (Fig. 5F). These results indicate that PTPRH promotes tumor progression by inhibiting apoptosis and enhancing angiogenesis through activation of VEGF signaling.

### PTPRH is regulated by RBM15-mediated m6A modification

RBM15, a core component of the m6A methyltransferase complex [28], functions as a “writer” by recruiting the methylation machinery to specific RNA sites and facilitating m6A deposition [13]. TCGA analysis revealed significantly elevated RBM15 expression in LUAD, and high RBM15 expression was associated with reduced OS (Supplementary Fig. S3A, B). Our previous work confirmed RBM15 overexpression in lung cancer and demonstrated its role in promoting both tumor progression and osimertinib resistance. In RBM15-overexpressing cells, global m6A levels were increased, with enrichment near stop codons [16].

**Fig. 6:**
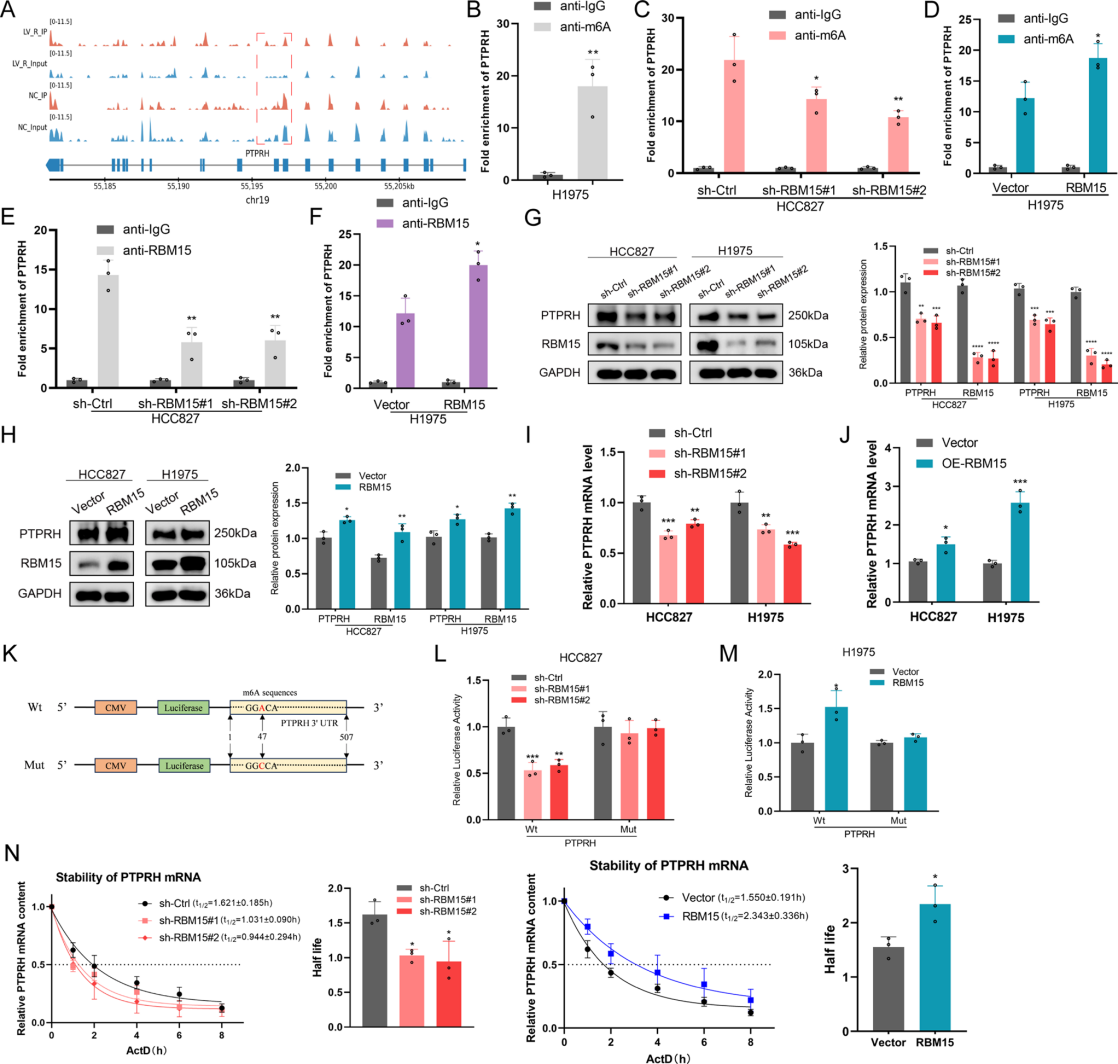
PTPRH is regulated by RBM15-mediated m6A methylation. (**A**) Differential m6A peaks on PTPRH identified by MeRIP-seq. (**B**) Validation of m6A enrichment on PTPRH by MeRIP-qPCR (*n* = 3 independent experiments, unpaired *t*-test), showing a fold change (anti-m6A vs. anti-IgG) of 18.0 (*p* = 0.0046). (**C**, **D**) m6A levels on PTPRH following RBM15 knockdown or overexpression (*n* = 3 independent experiments). (**C**) RBM15 knockdown reduced m6A levels (one-way ANOVA with Dunnett’s test; fold changes vs. sh-Ctrl: sh-RBM15#1 = 0.66, *p* = 0.041; sh-RBM15#2 = 0.43, *p* = 0.0078). (**D**) RBM15 overexpression increased m6A levels (unpaired *t*-test; fold change vs. Vector = 1.53, *p* = 0.0321). (**E**, **F**) Enrichment of PTPRH in RBM15 immunoprecipitates measured by RIP-qPCR (*n* = 3 independent experiments). (**E**) RBM15 knockdown reduced PTPRH enrichment (one-way ANOVA with Dunnett’s test; fold changes vs. sh-Ctrl: sh-RBM15#1 = 0.40, *p* = 0.0026; sh-RBM15#2 = 0.42, *p* = 0.003). (**F**) RBM15 overexpression increased enrichment (unpaired *t*-test; fold change vs. Vector = 1.64, *p* = 0.0027). (**G**, **H**) Western blot analysis of PTPRH expression following RBM15 modulation (*n* = 3 independent experiments, normalized to GAPDH), with representative blots shown. (**G**) RBM15 knockdown decreased both RBM15 and PTPRH protein levels in HCC827 and H1975 cells (one-way ANOVA with Dunnett’s test; PTPRH fold changes: 0.60–0.67; RBM15 fold changes: 0.21–0.30; all *p* < 0.01). (**H**) RBM15 overexpression increased both RBM15 and PTPRH protein levels (unpaired *t*-test; fold changes vs. Vector: HCC827, PTPRH = 1.25, *p* = 0.0104, RBM15 = 1.51, *p* = 0.0072; H1975, PTPRH = 1.25, *p* = 0.017, RBM15 = 1.40, *p* = 0.0013). (**I**, **J**) RT-qPCR analysis of *PTPRH* expression following RBM15 modulation (*n* = 3 independent experiments). (**I**) RBM15 knockdown reduced PTPRH levels in HCC827 and H1975 cells (one-way ANOVA with Dunnett’s test; fold changes vs. sh-Ctrl: 0.59–0.79; all *p* < 0.01). (**J**) RBM15 overexpression increased PTPRH levels (unpaired *t*-test; fold changes vs. Vector: HCC827 = 1.41, *p* = 0.0202; H1975 = 2.58, *p* = 0.0008). (**K**) Schematic diagram illustrating the predicted m6A methylation site within the *PTPRH* 3′UTR and the corresponding mutant construct. (**L**, **M**) Dual-luciferase reporter assays demonstrating m6A-dependent regulation of PTPRH by RBM15 (*n* = 3 independent experiments). (**L**) RBM15 knockdown significantly reduced wild-type (WT) reporter activity but had no effect on the mutant (Mut) reporter (one-way ANOVA with Dunnett’s test; WT: 0.53–0.59, *p* < 0.01; Mut: no significant change). (**M**) RBM15 overexpression increased WT reporter activity without affecting Mut reporter activity (unpaired *t*-test; WT = 1.52, *p* = 0.0277; Mut: not significant). (**N**) RNA stability of *PTPRH* transcripts following actinomycin D treatment in RBM15-silenced or -overexpressing cells (*n* = 3 independent experiments). Transcript half-life (t1/2) was derived from exponential decay curves. RBM15 knockdown reduced PTPRH stability (one-way ANOVA with Dunnett’s test; fold changes vs. sh-Ctrl: 0.64, *p* = 0.023; 0.58, *p* = 0.0126), whereas RBM15 overexpression increased transcript stability (unpaired *t*-test; fold change vs. Vector = 1.51, *p* = 0.0237)

Analysis of prior MeRIP-seq data revealed pronounced hypermethylation of m6A peaks within the PTPRH transcript (Fig. 6A), and the distribution of these peaks was supported by bioinformatic prediction using the SRAMP tool (Supplementary Fig. S3C). Subsequent validation by MeRIP-qPCR confirmed significant enrichment of m6A modifications on PTPRH mRNA (Fig. 6B). Moreover, RBM15 knockdown reduced, whereas overexpression increased, the m6A modification level of PTPRH mRNA (Fig. 6C, D). To determine whether RBM15 directly interacts with PTPRH transcripts, RNA immunoprecipitation assays were performed. PTPRH mRNA enrichment was significantly decreased upon RBM15 knockdown and increased upon RBM15 overexpression (Fig. 6E, F), indicating a direct interaction between RBM15 and PTPRH mRNA consistent with its role in site-specific m6A deposition.

Functionally, RBM15 silencing decreased both PTPRH mRNA and protein levels in HCC827 and H1975 cells (Fig. 6G, I), whereas RBM15 overexpression produced the opposite effect (Fig. 6H, J). Based on MeRIP-seq peak distribution and SRAMP prediction, a conserved RRACH motif (GGACA) was identified within the 3′UTR of PTPRH. To validate its functional relevance, site-directed mutagenesis was performed by substituting “ACA” with “CCA” (PTPRH-Mut construct; Fig. 6K). Dual-luciferase reporter assays showed that RBM15 knockdown significantly reduced luciferase activity of the wild-type (WT) reporter in HCC827 cells but had no effect on the mutant (Mut) reporter (Fig. 6L). Conversely, in H1975 cells, RBM15 overexpression enhanced WT reporter activity without affecting the Mut reporter (Fig. 6M), indicating that RBM15-dependent regulation of PTPRH is mediated through this m6A consensus site. Since m6A modifications are known to influence mRNA stability [29], we investigated whether RBM15‘s effect on PTPRH expression is mediated through this mechanism. Actinomycin D chase assays revealed that RBM15 knockdown significantly shortened the half-life of PTPRH transcripts, whereas overexpression extended it (Fig. 6N). These results demonstrate that RBM15 mediates m6A methylation of PTPRH mRNA and enhances its stability, thereby positively regulating PTPRH expression in NSCLC cells.

### RBM15 promotes malignant progression by stabilizing PTPRH

**Fig. 7:**
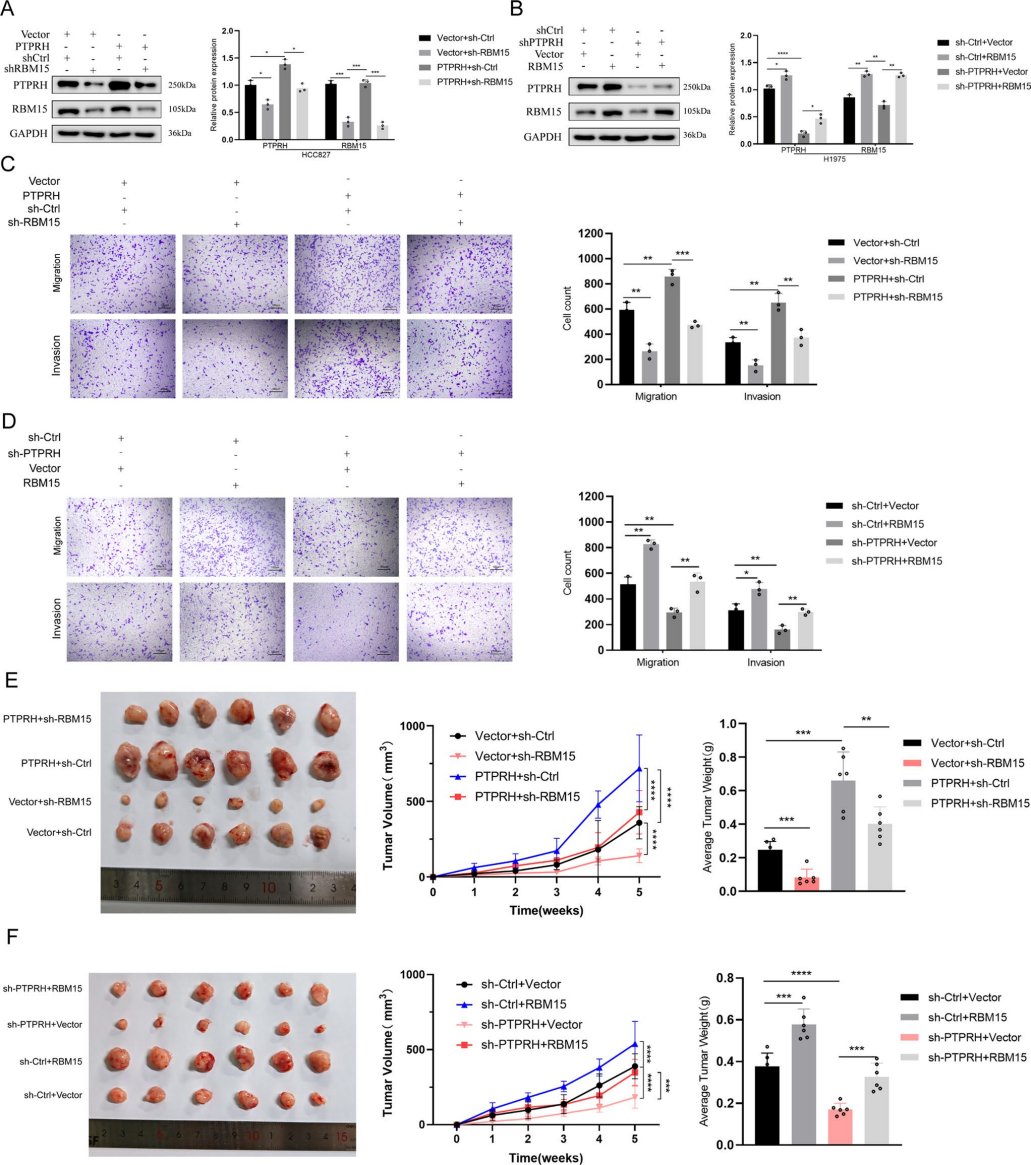
RBM15 promotes tumor progression by upregulating PTPRH. (**A**) Western blot analysis of PTPRH expression in HCC827 cells following RBM15 knockdown and/or PTPRH overexpression (*n* = 3 independent experiments, normalized to GAPDH), with representative blots shown. Data were analyzed by one-way ANOVA with Tukey’s test. PTPRH fold changes were as follows: Vector + sh-RBM15 vs. Vector + sh-Ctrl = 0.64 (*p* = 0.0038); PTPRH + sh-Ctrl vs. Vector + sh-Ctrl = 1.38 (*p* = 0.0026); PTPRH + sh-RBM15 vs. PTPRH + sh-Ctrl = 0.68 (*p* = 0.001). RBM15 fold changes were as follows: Vector + sh-RBM15 vs. Vector + sh-Ctrl = 0.32 (*p* < 0.0001); PTPRH + sh-RBM15 vs. PTPRH + sh-Ctrl = 0.25 (*p* < 0.0001). (**B**) Western blot analysis of PTPRH expression in H1975 cells following RBM15 overexpression and/or PTPRH knockdown (*n* = 3 independent experiments, normalized to GAPDH), with representative blots shown. Data were analyzed by one-way ANOVA with Tukey’s test. PTPRH fold changes were as follows: sh-Ctrl + RBM15 vs. sh-Ctrl + Vector = 1.26 (*p* = 0.0144); sh-PTPRH + Vector vs. sh-Ctrl + Vector = 0.19 (*p* < 0.0001); sh-PTPRH + RBM15 vs. sh-PTPRH + Vector = 2.40 (*p* = 0.0069). RBM15 fold changes were as follows: sh-Ctrl + RBM15 vs. sh-Ctrl + Vector = 1.50 (*p* < 0.0001); sh-PTPRH + RBM15 vs. sh-PTPRH + Vector = 1.80 (*p* < 0.0001). (**C**, **D**) Transwell assays evaluating migration and invasion following RBM15 and PTPRH modulation (*n* = 3 independent experiments). In HCC827 cells (**C**), one-way ANOVA with Tukey’s test showed that RBM15 knockdown reduced migration (0.44, *p* = 0.0003) and invasion (0.45, *p* = 0.0161) relative to Vector + sh-Ctrl, whereas PTPRH overexpression increased migration (1.45, *p* = 0.0012) and invasion (1.94, *p* = 0.0006). Co-transfection with sh-RBM15 reversed these effects (migration = 0.55, *p* < 0.0001; invasion = 0.57, *p* = 0.0013 vs. PTPRH + sh-Ctrl). In H1975 cells (**D**), RBM15 overexpression enhanced migration (1.60, *p* = 0.0003) and invasion (1.55, *p* = 0.0043), whereas PTPRH knockdown reduced migration (0.57, *p* = 0.0034) and invasion (0.52, *p* = 0.0089) relative to sh-Ctrl + Vector. Co-expression of RBM15 partially rescued these effects (migration = 1.51, *p* = 0.002; invasion = 1.81, *p* = 0.002 vs. sh-PTPRH + Vector). (**E**) Suppression of xenograft tumor growth by RBM15 knockdown in PTPRH-overexpressing cells (*n* = 6 mice per group). Tumor volume was measured weekly for 5 weeks and analyzed by two-way ANOVA with Tukey’s test. Fold changes at week 5 were as follows: Vector + sh-RBM15 vs. Vector + sh-Ctrl = 0.39 (*p* < 0.0001); PTPRH + sh-Ctrl vs. Vector + sh-Ctrl = 2.00 (*p* < 0.0001); PTPRH + sh-RBM15 vs. PTPRH + sh-Ctrl = 0.60 (*p* < 0.0001). Final tumor weights at necropsy were analyzed by one-way ANOVA with Tukey’s test, showing fold changes of 0.34 (*p* = 0.0041), 2.68 (*p* = 0.0011), and 0.61 (*p* = 0.0019), respectively. (**F**) Restoration of tumor growth by RBM15 overexpression in PTPRH-silenced cells (*n* = 6 mice per group). Tumor volume was measured weekly for 5 weeks and analyzed by two-way ANOVA with Tukey’s test. Fold changes at week 5 were as follows: sh-Ctrl + RBM15 vs. sh-Ctrl + Vector = 1.39 (*p* < 0.0001); sh-PTPRH + Vector vs. sh-Ctrl + Vector = 0.47 (*p* < 0.0001); sh-PTPRH + RBM15 vs. sh-PTPRH + Vector = 1.91 (*p* < 0.0001). Final tumor weights were analyzed by one-way ANOVA with Tukey’s test, showing fold changes of 1.53 (*p* < 0.0001), 0.47 (*p* < 0.0001), and 1.91 (*p* = 0.0012), respectively

In HCC827 cells, RBM15 knockdown reduced the expression of both RBM15 and PTPRH proteins and partially attenuated the upregulation of PTPRH induced by its overexpression (Fig. 7A). Conversely, in H1975 cells, RBM15 overexpression increased the levels of both proteins and partially rescued the suppressive effects of PTPRH silencing (Fig. 7B). Transwell assays further demonstrated that RBM15 knockdown significantly impaired cell migration and invasion, and the pro-migratory and pro-invasive effects induced by PTPRH overexpression were effectively reversed by co-transfection with sh-RBM15 (Fig. 7C). In contrast, RBM15 overexpression enhanced migratory and invasive capacities, partially restoring the inhibitory effects caused by PTPRH knockdown (Fig. 7D).

We next examined whether this regulatory axis influences apoptosis. The anti-apoptotic effect of PTPRH overexpression was diminished by RBM15 knockdown, whereas the pro-apoptotic effect of PTPRH silencing was mitigated by RBM15 overexpression (Supplementary Fig. S3D). Consistent results were observed in nude mouse xenograft models. RBM15 knockdown attenuated the tumor-promoting effect of PTPRH overexpression, while RBM15 overexpression reversed the tumor suppression induced by PTPRH knockdown (Fig. 7E, F).

### IGF2BP2 recognizes m6A-modified PTPRH and enhances its stability

**Fig. 8:**
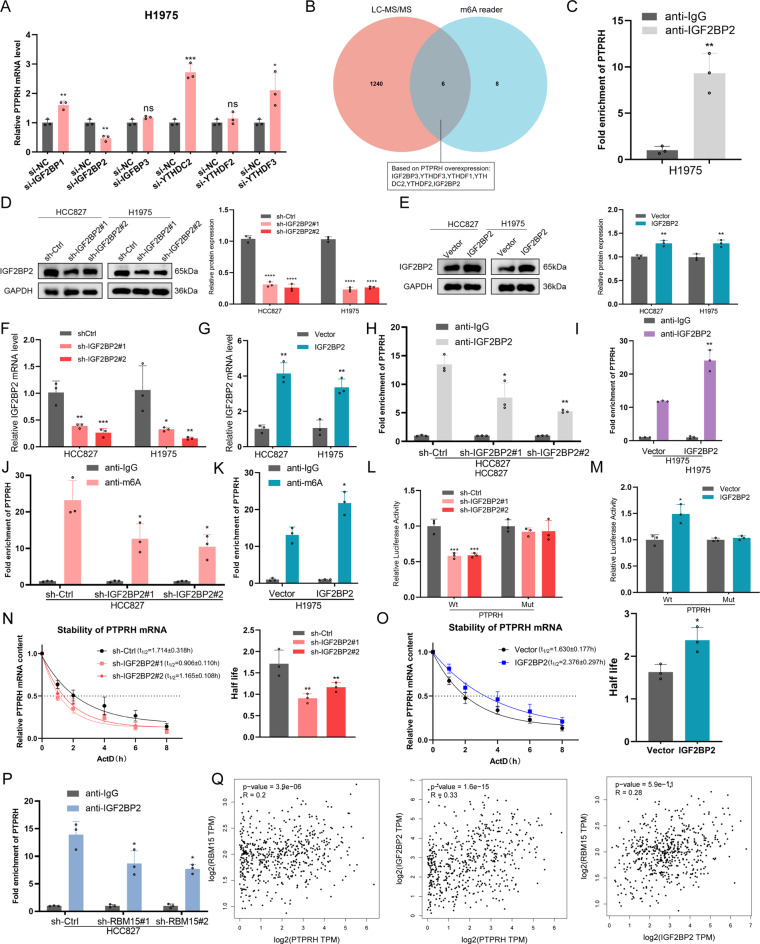
IGF2BP2 recognizes m6A-modified PTPRH mRNA. (**A**) qPCR analysis of *PTPRH* expression following knockdown of m6A reader proteins. (**B**) Venn diagram showing overlap between proteins identified by LC–MS/MS and known m6A readers. (**C**) RIP-qPCR confirming binding of IGF2BP2 to *PTPRH* mRNA (*n* = 3 independent experiments, unpaired *t*-test), with a fold change (anti-IGF2BP2 vs. anti-IgG) of 9.3 (*p* = 0.0027). (**D**, **E**) Protein expression of IGF2BP2 following knockdown or overexpression in HCC827 and H1975 cells (*n* = 3 independent experiments, normalized to GAPDH), with representative blots shown. (**D**) Knockdown significantly reduced IGF2BP2 protein levels (one-way ANOVA with Dunnett’s test; fold changes vs. sh-Ctrl: HCC827 = 0.31 and 0.26; H1975 = 0.23 and 0.25; all *p* < 0.0001). (**E**) Overexpression increased IGF2BP2 protein levels (unpaired *t*-test; fold changes vs. Vector: HCC827 = 1.29, *p* = 0.0024; H1975 = 1.30, *p* = 0.007). (**F**, **G**) mRNA expression of *IGF2BP2* following knockdown or overexpression (*n* = 3 independent experiments). (**F**) Knockdown reduced *IGF2BP2* mRNA levels (one-way ANOVA with Dunnett’s test; fold changes vs. sh-Ctrl: HCC827 = 0.38 and 0.26; H1975 = 0.32 and 0.16; all *p* < 0.05). (**G**) Overexpression increased *IGF2BP2* mRNA levels (unpaired *t*-test; fold changes vs. Vector: HCC827 = 4.14, *p* = 0.0011; H1975 = 3.36, *p* = 0.0037). (**H**, **I**) Enrichment of *PTPRH* mRNA in IGF2BP2 immunoprecipitates measured by RIP-qPCR (*n* = 3 independent experiments). (**H**) IGF2BP2 knockdown reduced PTPRH enrichment (one-way ANOVA with Dunnett’s test; fold changes vs. sh-Ctrl: 0.57, *p* = 0.0115; 0.39, *p* = 0.0021). (**I**) IGF2BP2 overexpression increased enrichment (unpaired *t*-test; fold change vs. Vector = 2.03, *p* = 0.0027). (**J**, **K**) Relative abundance of m6A-modified *PTPRH* transcripts following IGF2BP2 knockdown or overexpression (*n* = 3 independent experiments). (**J**) Knockdown reduced m6A-modified PTPRH levels (one-way ANOVA with Dunnett’s test; fold changes vs. sh-Ctrl: 0.54, *p* = 0.0436; 0.46, *p* = 0.0208). (**K**) Overexpression increased m6A-modified PTPRH levels (unpaired *t*-test; fold change vs. Vector = 1.66, *p* = 0.0167). (**L**, **M**) Dual-luciferase reporter assays demonstrating m6A-dependent regulation of PTPRH by IGF2BP2 (*n* = 3 independent experiments). (**L**) IGF2BP2 knockdown significantly reduced wild-type (WT) reporter activity but had no effect on the mutant (Mut) reporter (one-way ANOVA with Dunnett’s test; WT: 0.58–0.59, *p* = 0.0004; Mut: not significant). (**M**) IGF2BP2 overexpression increased WT reporter activity without affecting Mut reporter activity (unpaired *t*-test; WT = 1.49, *p* = 0.0145; Mut: not significant). (**N**, **O**) RNA stability assays showing that IGF2BP2 knockdown decreased, whereas overexpression increased, the stability of *PTPRH* transcripts. (**P**) Effect of RBM15 knockdown on the interaction between *IGF2BP2* and *PTPRH* (*n* = 3 independent experiments; one-way ANOVA with Dunnett’s test; fold changes vs. sh-Ctrl: 0.62, *p* = 0.0311; 0.55, *p* = 0.0153). (**Q**) Correlation analysis of *RBM15*, *IGF2BP2*, and *PTPRH* expression in TCGA datasets

Among the m6A “reader” proteins, members of the YTH and IGF2BP families play critical roles in regulating RNA fate [17, 30]. To identify the reader responsible for modulating PTPRH, we designed siRNAs targeting representative members of both families. Silencing experiments revealed that IGF2BP2 significantly influenced PTPRH expression (Fig. 8A). To further validate this interaction, RNA pull-down assays were performed using biotinylated PTPRH probes. Electrophoresis and silver staining revealed distinct bands enriched in the sense probe group (Supplementary Fig. S3E), which were analyzed by LC-MS/MS. Comparison with known m6A readers again identified IGF2BP2 (Fig. 8B). As an RNA-binding protein that regulates mRNA stability and translation [31], IGF2BP2 was confirmed to directly interact with PTPRH mRNA by RIP-qPCR (Fig. 8C). TCGA analysis showed high IGF2BP2 expression in LUAD, with elevated levels associated with worse survival (Supplementary Fig. S3F, G). Consistent with these findings, immunohistochemistry of clinical samples showed increased IGF2BP2 expression in tumor tissues compared with adjacent normal tissues (Supplementary Fig. S3H).

To functionally characterize this interaction, HCC827 and H1975 cells were transduced with validated shRNAs targeting IGF2BP2 or an IGF2BP2 overexpression construct (Fig. 8D–G). IGF2BP2 knockdown reduced the enrichment of PTPRH mRNA, whereas overexpression increased its enrichment (Fig. 8H, I). Similarly, the relative abundance of m6A-modified PTPRH mRNA decreased following IGF2BP2 silencing and increased upon overexpression (Fig. 8J, K). To determine whether this regulation is m6A-dependent, a dual-luciferase reporter assay was employed. In HCC827 cells, IGF2BP2 knockdown significantly reduced the activity of the wild-type (WT) reporter but had no effect on the mutant (Mut) reporter (Fig. 8L). Conversely, in H1975 cells, IGF2BP2 overexpression enhanced WT reporter activity without affecting Mut reporter activity (Fig. 8M), indicating that IGF2BP2-mediated regulation of PTPRH depends on an intact m6A consensus site within its 3′UTR. RNA stability assays further demonstrated that IGF2BP2 depletion significantly shortened the half-life of PTPRH transcripts, whereas overexpression prolonged their stability (Fig. 8N, O). RIP-qPCR analysis showed that RBM15 knockdown weakened the interaction between IGF2BP2 and PTPRH mRNA (Fig. 8P). In addition, TCGA data analysis revealed a strong positive correlation among RBM15, IGF2BP2, and PTPRH expression levels (Fig. 8Q). These findings demonstrate that IGF2BP2 recognizes m6A-modified PTPRH transcripts and enhances their stability, thereby contributing to the regulation of PTPRH expression in NSCLC.

### IGF2BP2 cooperates with PTPRH to promote malignant phenotypes

**Fig. 9:**
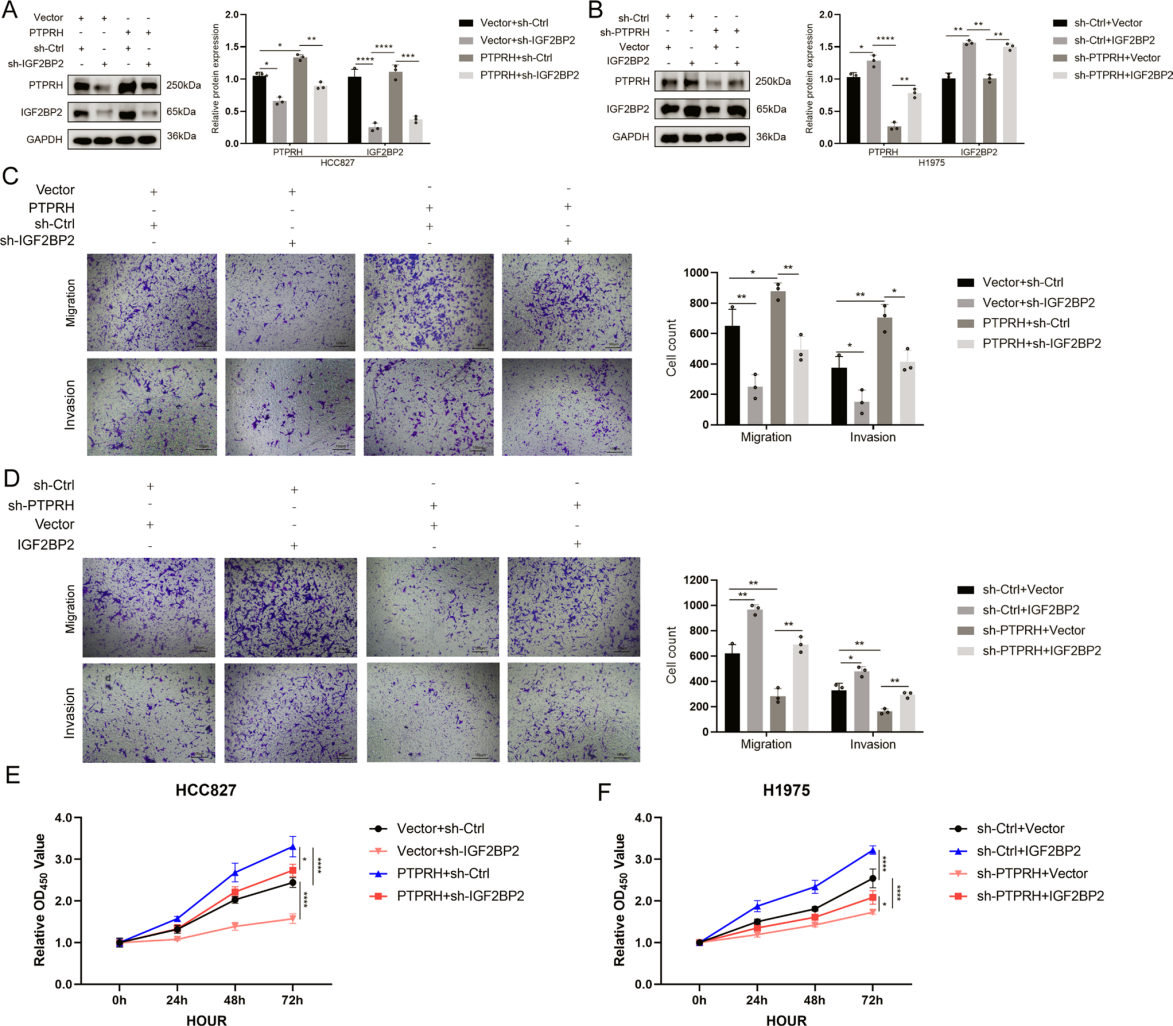
IGF2BP2 promotes tumor progression by upregulating PTPRH. (**A**) Western blot analysis of PTPRH expression in HCC827 cells following IGF2BP2 knockdown and/or PTPRH overexpression (*n* = 3 independent experiments, normalized to GAPDH), with representative blots shown. Data were analyzed by one-way ANOVA with Tukey’s test. PTPRH fold changes were as follows: Vector + sh-IGF2BP2 vs. Vector + sh-Ctrl = 0.65 (*p* = 0.020); PTPRH + sh-Ctrl vs. Vector + sh-Ctrl = 1.33 (*p* = 0.0326); PTPRH + sh-IGF2BP2 vs. PTPRH + sh-Ctrl = 0.68 (*p* = 0.0061). IGF2BP2 fold changes were as follows: Vector + sh-IGF2BP2 vs. Vector + sh-Ctrl = 0.25 (*p* < 0.0001); PTPRH + sh-IGF2BP2 vs. PTPRH + sh-Ctrl = 0.34 (*p* = 0.0037). (**B**) Western blot analysis of PTPRH expression in H1975 cells following IGF2BP2 overexpression and/or PTPRH knockdown (*n* = 3 independent experiments, normalized to GAPDH), with representative blots shown. Data were analyzed by one-way ANOVA with Tukey’s test. PTPRH fold changes were as follows: sh-Ctrl + IGF2BP2 vs. sh-Ctrl + Vector = 1.28 (*p* = 0.0111); sh-PTPRH + Vector vs. sh-Ctrl + Vector = 0.25 (*p* < 0.0001); sh-PTPRH + IGF2BP2 vs. sh-PTPRH + Vector = 2.96 (*p* = 0.001). IGF2BP2 fold changes were as follows: sh-Ctrl + IGF2BP2 vs. sh-Ctrl + Vector = 1.56 (*p* = 0.031); sh-PTPRH + IGF2BP2 vs. sh-PTPRH + Vector = 1.50 (*p* = 0.0271). (**C**, **D**) Transwell assays evaluating migration and invasion following IGF2BP2 and PTPRH modulation (*n* = 3 independent experiments, one-way ANOVA with Tukey’s test). In HCC827 cells (**C**), IGF2BP2 knockdown reduced migration (0.38, *p* = 0.0018) and invasion (0.40, *p* = 0.0338) relative to Vector + sh-Ctrl, whereas PTPRH overexpression increased migration (1.35, *p* = 0.0436) and invasion (1.88, *p* = 0.0051). Co-transfection with sh-IGF2BP2 reversed these effects (migration = 0.58, *p* = 0.0023; invasion = 0.59, *p* = 0.0084 vs. PTPRH + sh-Ctrl). In H1975 cells (**D**), IGF2BP2 overexpression enhanced migration (1.56, *p* = 0.0054) and invasion (1.46, *p* = 0.0052), whereas PTPRH knockdown reduced migration (0.46, *p* = 0.0061) and invasion (0.49, *p* = 0.0027) relative to sh-Ctrl + Vector. Co-expression of IGF2BP2 partially rescued these effects (migration = 2.43, *p* = 0.0053; invasion = 1.83, *p* = 0.0015 vs. sh-PTPRH + Vector). (**E**, **F**) CCK-8 assays assessing cell proliferation following IGF2BP2 and PTPRH modulation (*n* = 3 independent experiments, two-way ANOVA with Tukey’s test). At 72 h, in HCC827 cells (**E**), IGF2BP2 knockdown reduced proliferation (0.64, *p* < 0.0001), whereas PTPRH overexpression increased proliferation (1.35, *p* < 0.0001), and co-transfection with sh-IGF2BP2 attenuated this effect (0.83, *p* = 0.023 vs. PTPRH + sh-Ctrl). In H1975 cells (**F**), IGF2BP2 overexpression enhanced proliferation (1.27, *p* < 0.0001), PTPRH knockdown reduced proliferation (0.68, *p* < 0.0001), and co-expression of IGF2BP2 partially restored proliferative capacity (1.21, *p* = 0.0253 vs. sh-PTPRH + Vector)

To define the role of IGF2BP2 in PTPRH-driven tumor progression, a series of rescue experiments was performed. In HCC827 cells, IGF2BP2 knockdown partially abrogated the increase in PTPRH expression induced by PTPRH overexpression (Fig. 9A). Conversely, in H1975 cells, IGF2BP2 overexpression partially restored PTPRH protein levels reduced by PTPRH knockdown (Fig. 9B). The functional significance of this interaction was further evaluated using migration and invasion assays. In HCC827 cells, PTPRH overexpression markedly enhanced migratory and invasive capacities, whereas co-transfection with sh-IGF2BP2 reversed these effects (Fig. 9C). In contrast, PTPRH silencing in H1975 cells impaired migration and invasion, and this phenotype was partially rescued by co-overexpression of IGF2BP2 (Fig. 9D). The cooperative effects on cell proliferation were assessed using the CCK-8 assay. In HCC827 cells, PTPRH overexpression significantly increased cell proliferation, whereas co-transfection with sh-IGF2BP2 attenuated this effect (Fig. 9E). Similarly, PTPRH knockdown in H1975 cells reduced proliferative capacity, which was partially restored by IGF2BP2 overexpression (Fig. 9F). These results demonstrate that IGF2BP2 cooperates with PTPRH to promote malignant phenotypes and, together with RBM15, contributes to the maintenance of PTPRH expression and its oncogenic functions in NSCLC.

## Discussion

Lung cancer remains the malignancy with the highest incidence and mortality worldwide [2]. Despite notable advances in targeted therapy and immunotherapy [32], the survival of patients with advanced disease remains poor [33], underscoring the urgent need for a deeper understanding of its pathogenesis. Increasing evidence indicates that epigenetic modifications play a pivotal role in shaping tumor biology, influencing processes such as proliferation, immune evasion, and metastasis, and that their dysregulation can profoundly affect tumor progression and therapeutic response [5]. Among these modifications, m6A methylation, the most abundant internal modification of eukaryotic mRNA, has attracted considerable attention. Multiple studies have demonstrated its role in driving tumor proliferation, migration, and invasion [34], highlighting the importance of dissecting its functions in lung cancer development.

PTPRH, a member of the PTP family, regulates phosphorylation homeostasis in coordination with RTKs and has been implicated in tumorigenesis [20]. While earlier studies reported oncogenic functions of PTPRH [23, 35], its potential connection to epigenetic regulation had not been described. Consistent with prior observations [26, 27], our analyses of TCGA and GEO datasets confirmed that PTPRH is significantly upregulated in lung cancer and associated with poor prognosis. These findings were further validated in paired clinical samples, where RT-qPCR and immunohistochemical analyses confirmed elevated PTPRH expression in tumor tissues relative to adjacent normal tissues. These results support the role of PTPRH as a clinically relevant oncogene and suggest its potential utility as both an independent prognostic biomarker and a therapeutic target in NSCLC.

Functional experiments further established that PTPRH promotes malignant progression. Its elevated expression across multiple cell lines mirrored bioinformatic and clinical findings. Gain- and loss-of-function assays demonstrated that PTPRH enhances proliferation, migration, and invasion, findings corroborated in xenograft models. Bioinformatic analyses indicated enrichment of apoptosis and angiogenesis pathways, which were subsequently validated experimentally: PTPRH suppressed apoptosis and promoted angiogenesis through modulation of the VEGF pathway. Given the central role of angiogenesis in solid tumor progression [36, 37] and the established efficacy of anti-angiogenic strategies in lung cancer [38, 39], these findings underscore the oncogenic potential of PTPRH.

Growing evidence supports m6A modification as a key driver of tumor biology [40, 41]. Building on our earlier work showing that RBM15 is overexpressed in lung cancer and promotes progression and drug resistance via m6A-dependent mechanisms [16], we examined its regulation of PTPRH. RBM15, a member of the SPEN family, recruits the methyltransferase complex to specific RNA sites [13] and has been implicated in promoting oncogenesis by stabilizing oncogenic transcripts [42]. In the present study, MeRIP-qPCR confirmed that PTPRH transcripts are enriched in m6A modifications, consistent with prior sequencing data. RBM15 overexpression enhanced both m6A modification and stability of PTPRH transcripts, whereas RBM15 knockdown exerted the opposite effect. Rescue experiments confirmed that RBM15 partially reverses the oncogenic functions of PTPRH in vitro and in vivo, establishing it as a critical upstream regulator.

**Fig. 10:**
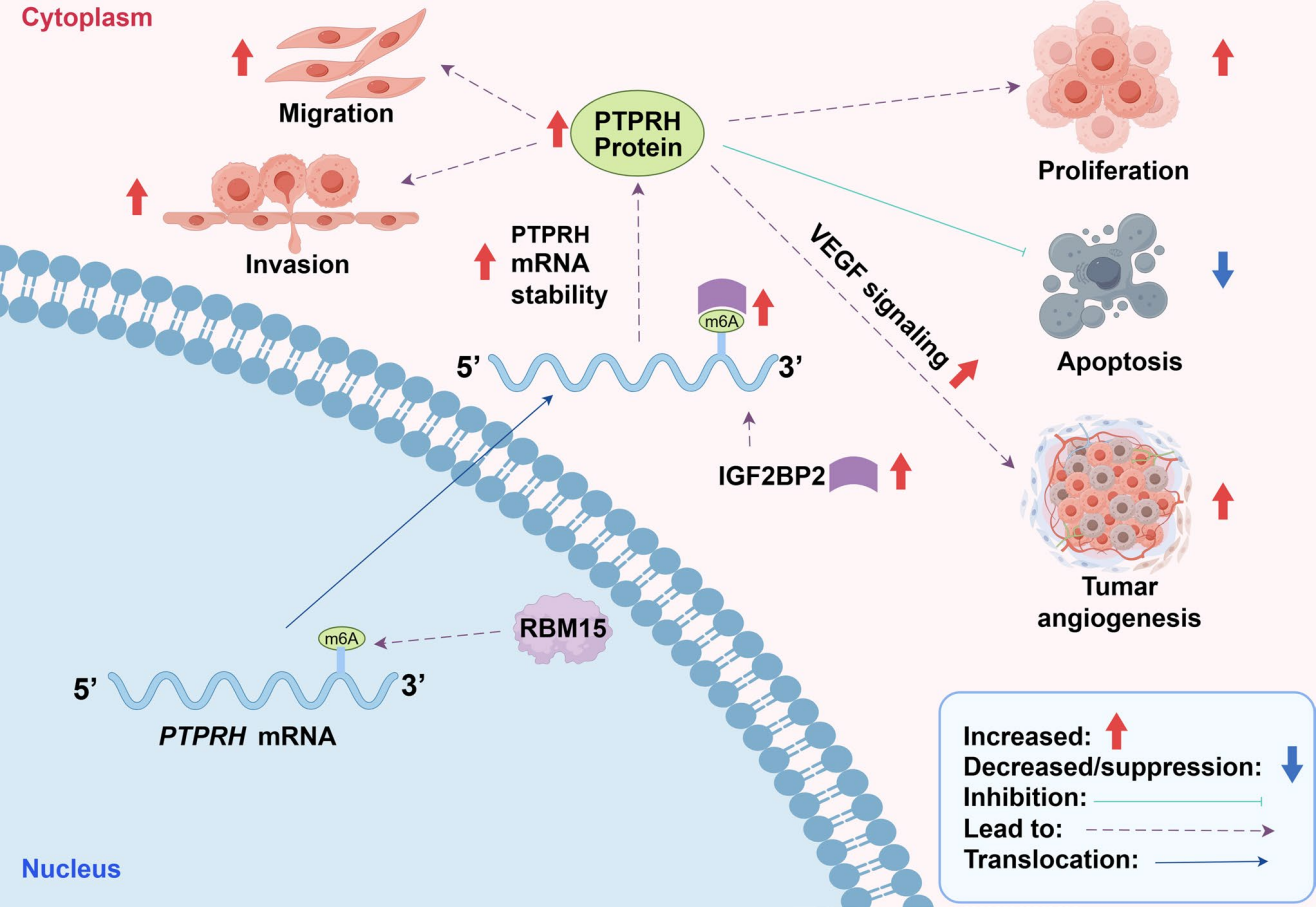
Schematic illustration of the RBM15/IGF2BP2–PTPRH regulatory axis in tumor progression. This model depicts how RBM15-mediated m6A modification and IGF2BP2-dependent recognition cooperatively enhance the stability of *PTPRH* mRNA, thereby promoting oncogenesis through increased cell proliferation, suppression of apoptosis, and stimulation of angiogenesis

The biological consequences of m6A modifications are executed by reader proteins [41]. Using RNA pull-down and LC-MS/MS analysis, we identified IGF2BP2 as the primary reader recognizing m6A-modified PTPRH mRNA. IGF2BP2 is known to regulate RNA stability and translation [31] and has previously been associated with poor prognosis and tumor angiogenesis in lung cancer [43]. Consistent with these reports, we observed high IGF2BP2 expression in lung cancer samples, where it correlated with worse survival. RIP-qPCR confirmed direct binding of IGF2BP2 to PTPRH mRNA, while functional assays showed that IGF2BP2 expression modulates both the m6A level and stability of PTPRH transcripts. Notably, RBM15 knockdown weakened the interaction between IGF2BP2 and PTPRH, indicating that RBM15-mediated methylation is a prerequisite for IGF2BP2 recognition. Rescue experiments further demonstrated that IGF2BP2 partially reverses the effects of PTPRH modulation, establishing a cooperative RBM15/IGF2BP2–PTPRH regulatory axis (Fig. 10).

In summary, we identify PTPRH as a key oncogene in lung cancer and demonstrate that its expression is regulated by RBM15-mediated m6A modification and IGF2BP2-dependent stabilization. This RBM15/IGF2BP2–PTPRH axis promotes proliferation, invasion, resistance to apoptosis, and angiogenesis, thereby driving malignant progression. Despite these findings, several important questions remain. First, the direct contribution of the METTL3/14 writer complex to this regulatory axis has yet to be formally established and warrants further investigation, for example, through loss-of-function or catalytic-dead mutant approaches. Second, given that both RBM15 and IGF2BP2 broadly regulate m6A-modified transcripts, the oncogenic effects of this axis are likely not restricted to PTPRH alone. Thus, the PTPRH pathway described here represents a central, but not exclusive, mechanism underlying RBM15/IGF2BP2-driven NSCLC progression. In addition, the downstream signaling mechanisms through which this axis promotes angiogenesis require further clarification. Although our functional rescue experiments demonstrate a clear dependence of VEGF-related phenotypes on PTPRH, the direct dephosphorylation substrates mediating this effect remain undefined. Based on the enrichment of VEGF signaling pathways identified by GSEA (Fig. 4B, C) and prior evidence implicating PTPRH in the regulation of the PI3K/AKT/mTOR pathway [27], we speculate that PTPRH may indirectly modulate the HIF-1α/VEGF axis through this signaling cascade. While our study delineates a novel upstream regulatory mechanism, identification of the direct downstream substrates of PTPRH remains a critical next step. Future studies incorporating phosphoproteomic approaches will be essential to define these targets and further elucidate the molecular mechanisms underlying PTPRH-driven tumor progression.

Beyond these cell-intrinsic mechanisms, our findings may have broader implications for the tumor immune microenvironment, which plays a critical role in cancer progression and therapeutic response. The observation that PTPRH enhances angiogenesis via VEGF signaling while suppressing apoptosis is consistent with emerging evidence that metabolic and signaling reprogramming in cancer cells can influence immune cell recruitment and function [44]. Molecular stratification strategies, such as the DNB-based subtyping approach reported by Zhang et al., which captures differences in immune contexture, may facilitate future patient selection for immunotherapy [45]. In addition, cloud-based integrative platforms, such as that described by Ke et al., provide a practical framework for validating the RBM15/IGF2BP2–PTPRH axis in large-scale, multi-omic cohorts and for exploring its potential interactions with immune checkpoint pathways [46]. Collectively, these observations suggest that targeting the RBM15/IGF2BP2–PTPRH axis may not only suppress tumor-intrinsic oncogenic processes but also modulate the immune landscape. This provides a rationale for combining m6A-targeted strategies with immunotherapy in NSCLC. Our findings highlight the RBM15/IGF2BP2–PTPRH axis as a potential therapeutic target in lung cancer, expanding current understanding of epitranscriptomic regulation in tumor biology.

## Conclusion

This study identifies PTPRH as a clinically relevant oncogene in lung cancer and demonstrates that its dysregulation promotes proliferation, invasion, resistance to apoptosis, and angiogenesis. Mechanistically, RBM15-mediated m6A modification and IGF2BP2 recognition cooperatively stabilize PTPRH transcripts, establishing a novel RBM15/IGF2BP2–PTPRH regulatory axis. These findings not only expand current understanding of epitranscriptomic regulation in tumor biology but also highlight this pathway as a promising therapeutic target. Future research should focus on defining the downstream substrates of PTPRH and exploring pharmacologic strategies to disrupt this axis, thereby providing new avenues for precision therapy in lung cancer.

## Electronic supplementary material

Below is the link to the electronic supplementary material.

Supplementary material 1

Supplementary material 2

Supplementary material 3

Supplementary material 4

Supplementary material 5

## Data Availability

The data supporting the findings of this study are available from the corresponding author upon reasonable request.

## Abbreviations

NSCLC: Non-small cell lung cancer
PTPRH: Protein tyrosine phosphatase receptor type H
RBM15: RNA-binding motif protein 15
IGF2BP2: Insulin-like growth factor 2 mRNA-binding protein 2
m6A: N6-methyladenosine
LUAD: Lung adenocarcinoma
PTP: Protein tyrosine phosphatases
RTK: Receptor tyrosine kinase
MeRIP-qPCR: m6A-RNA immunoprecipitation–qPCR
LC-MS/MS: Liquid chromatography–tandem mass spectrometry
VEGF: Vascular Endothelial Growth Factor
