# Supplementary material for: RBM15/IGF2BP2–PTPRH m6A regulatory axis in non-small cell lung cancer
Source: Cell Oncol (Dordr). 2026 May 11;49(3):85. doi: 10.1007/s13402-026-01217-2 (PMC13250017; doi:10.1007/s13402-026-01217-2)
Supplement: Supplementary file 1 — Supplementary material 1 [file 13402_2026_1217_MOESM1_ESM.docx]

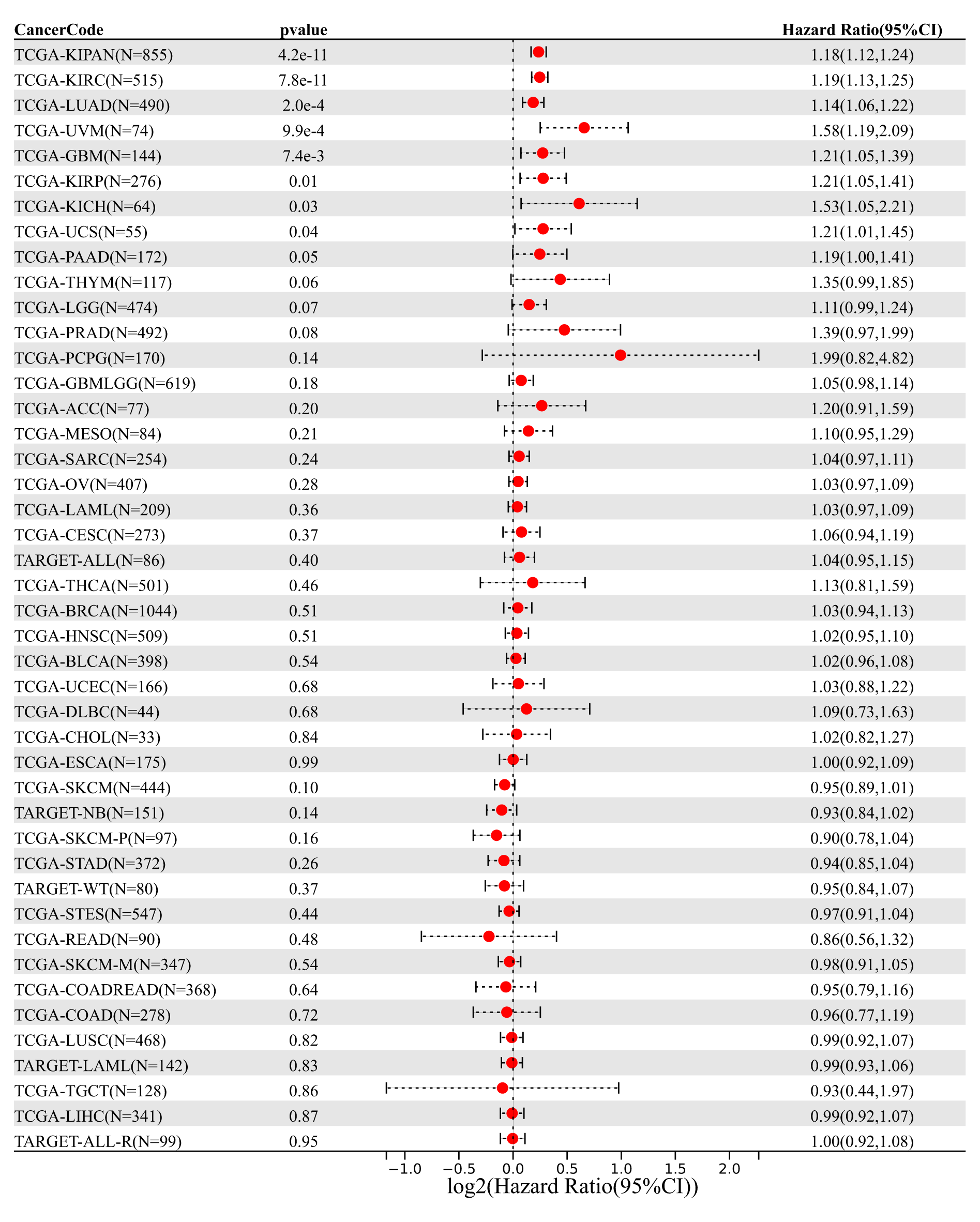


**Figure S1.** PTPRH expression was significantly associated with poorer overall survival in multiple cancer types, most notably in kidney cancers (KIPAN, KIRC) and LUAD.


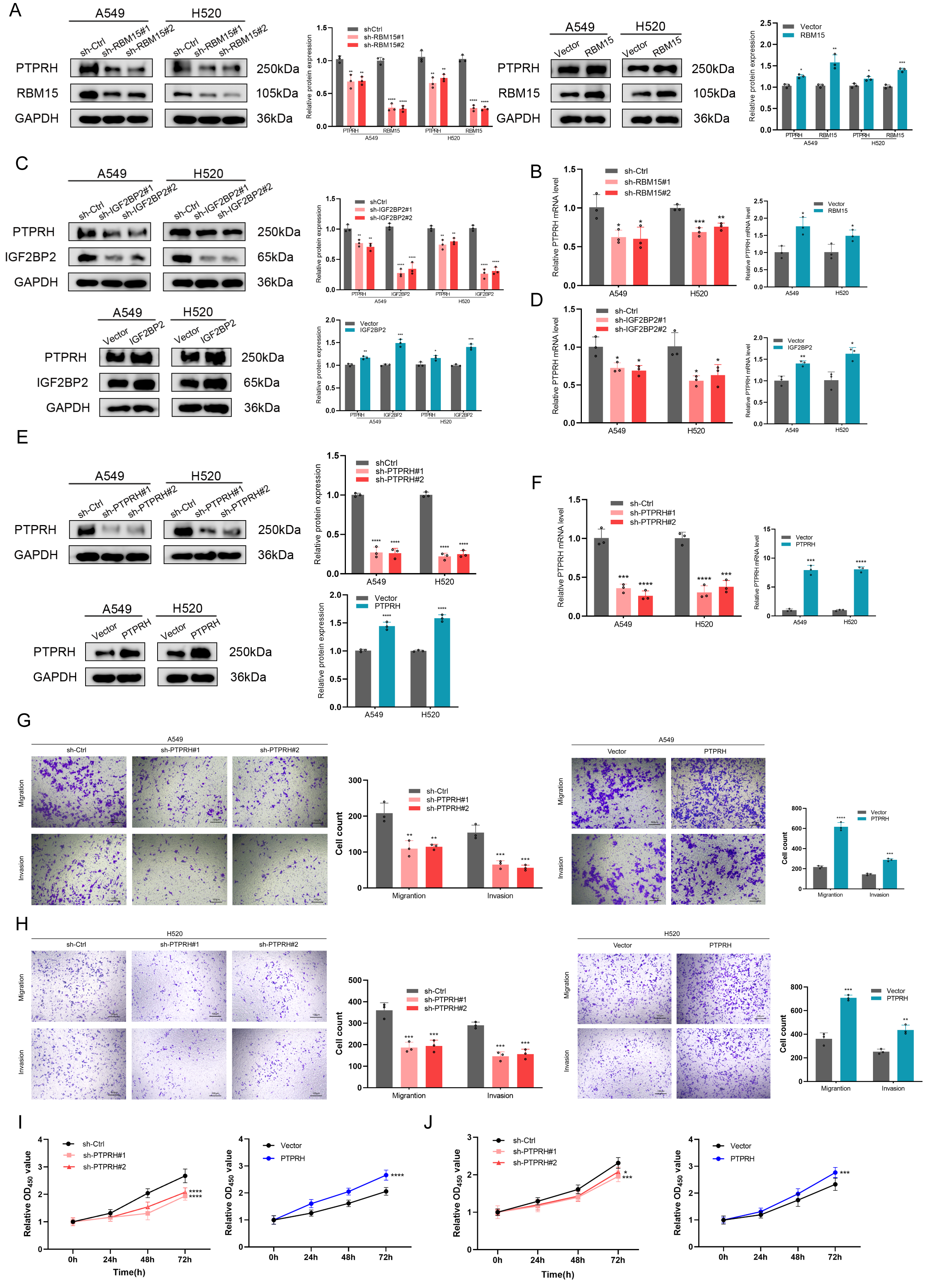


**Figure S2.** Pro-tumorigenic effects of PTPRH in KRAS-mutant LUAD (A549) and LUSC (H520) cell lines. **(A)** Western blot analysis of PTPRH expression after RBM15 modulation (n = 3 independent experiments, normalized to GAPDH). Representative blots are shown. (A, left) Knockdown effects in A549 and H520 cells (one-way ANOVA with Dunnett's test), (A, right) Overexpression effects (unpaired t-test). **(B)** RT-qPCR analysis of PTPRH expression after RBM15 modulation (n = 3 independent experiments). Knockdown effects in A549 and H520 cells (one-way ANOVA with Dunnett's test), Overexpression effects (unpaired t-test). **(C)** Western blot analysis of PTPRH expression after IGF2BP2 modulation (n = 3 independent experiments, normalized to GAPDH). Representative blots are shown. (C, upper) Knockdown effects in A549 and H520 cells (one-way ANOVA with Dunnett's test), (C, lower) Overexpression effects (unpaired t-test). **(D)** RT-qPCR analysis of PTPRH expression after IGF2BP2 modulation (n = 3 independent experiments). Knockdown effects in A549 and H520 cells (one-way ANOVA with Dunnett's test), Overexpression effects (unpaired t-test). **(E)** Western blot validation of PTPRH knockdown and overexpression. (n = 3 independent experiments, normalized to GAPDH). Representative blots are shown. (E, upper) Knockdown effects in A549 and H520 cells (one-way ANOVA with Dunnett's test), (E, lower) Overexpression effects (unpaired t-test). **(F)** RT-qPCR validation of PTPRH knockdown and overexpression. (n = 3 independent experiments). Knockdown effects in A549 and H520 cells (one-way ANOVA with Dunnett's test), Overexpression effects (unpaired t-test). **(G, H)** Cell migration and invasion assessed by Transwell assay. (G) A549 cells. Knockdown effects (one-way ANOVA with Dunnett's test), Overexpression effects (unpaired t-test). (H) H520 cells. Knockdown effects (one-way ANOVA with Dunnett's test), Overexpression effects (unpaired t-test). (I, J) Cell proliferation measured by CCK-8 assay. (n = 3 independent experiments). (I, left) A549 Knockdown effects (two-way ANOVA with Tukey's test), (I, right) A549 Overexpression effects (two-way ANOVA with Sidak's test). (J, right) H520 Overexpression effects (two-way ANOVA with Sidak's test).


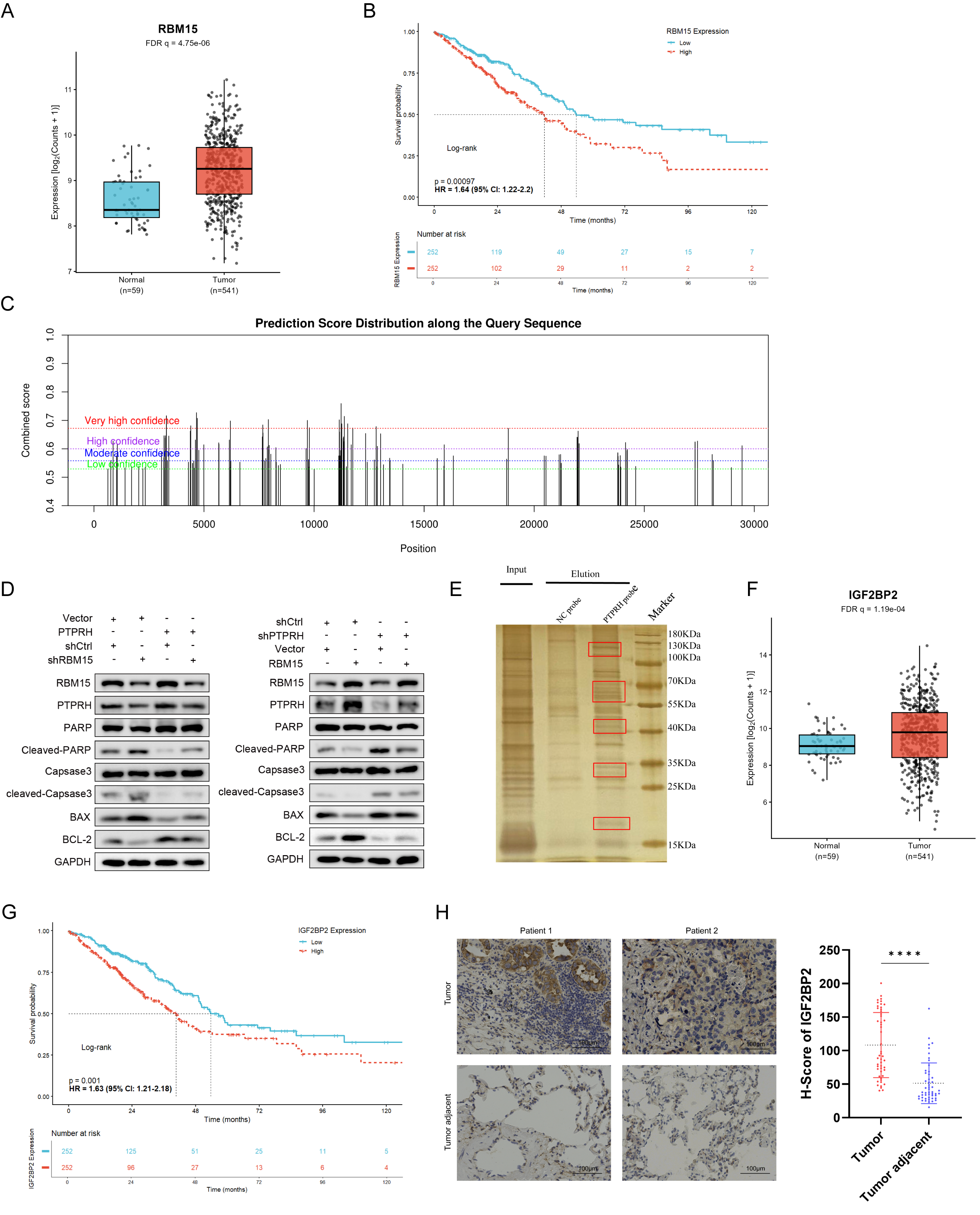


**Figure S3. (A)** Expression of RBM15 by TCGA transcriptomic data (Normal=59, Tumor=541, log2FC = 0.35, 95% CI: 0.06–0.63, FDR q = 4.75e-06). **(B)** Kaplan–Meier survival analysis of overall survival (OS) according to PTPRH expression. (n_high = 252, n_low = 252). The cutoff value was determined by median expression (log-rank test). HR = 1.64, 95% CI: 1.24–2.2, p = 0.00097. The number of patients at risk is shown below the plot. **(C)** In silico prediction of m6A methylation sites on the PTPRH transcript (or protein/cDNA sequence) using SRAMP. The prediction score (ranging from 0.4 to 1.0) is plotted against the nucleotide position. Peaks above the confidence thresholds indicate putative m6A sites, with colors representing different confidence levels: very high (red), high (purple), moderate (blue), and low (green). **(D)** The anti-apoptotic effect of PTPRH overexpression was diminished by RBM15 knockdown, whereas the pro-apoptotic effect of PTPRH silencing was mitigated by RBM15 overexpression. **(E)** Identification of proteins that bind to the PTPRH RNA probe by RNA pull-down assay. Specific binding proteins were visualized by silver staining. **(F)** Expression of IGF2BP2 by TCGA transcriptomic data (Normal=59, Tumor=541, log2FC = 0.83, 95% CI: 0.03–1.63, FDR q = 1e-04). **(G)** Kaplan–Meier survival analysis of overall survival (OS) according to PTPRH expression. (n_high = 252, n_low = 252). The cutoff value was determined by median expression (log-rank test). HR = 1.63, 95% CI: 1.21–2.18, p = 0.001. The number of patients at risk is shown below the plot. **(H)** IHC staining of IGF2BP2 in LUAD and adjacent tissues. (scale bar: 100 μm), Quantification from n = 50 paired samples, with 3 random fields analyzed per sample. PTPRH: Fold change = 2.11, p < 0.0001 (paired t-test).

| Sequences for shRNAs and siRNAs | | 5'-3' |
| --- | --- | --- |
| Sequences for shRNAs | | |
| shPTPRH#1 | | GCAGAACAGAGACTCGAAA |
| shPTPRH#2 | | CACCGGGACGTTGTACAA |
| shRBM15#1 | | GCCTGTTTCATGAGTTCAAAC |
| shRBM15#2 | | GGACCTTTATCCTGACTCT |
| shIGF2BP2#1 | | AGTGAAGCTGGAAGCGCATAT |
| shIGF2BP2#2 | | TTCCCGCATCATCACTCTTAT |
| Sequences for siRNAs | | |
| IGF2BP1 | | GCUCCCUAUAGCUCCUUUATT |
| IGF2BP2 | | AUAUACAACCCGGAAAGAATT |
| IGF2BP3 | | GGCUCAGGGAAGAAUUUAUTT |
| YTHDF2 | | CCUACCAGAUGCAAUGUUUTT |
| YTHDF3 | | ATGGATTAAATCAGTATCTAA |
| YTHDC2 | | GCGACUCAACAAUGGCAUATT |
| HNRNPA2B1 | | GAGGUGGUUAUGACAACUATT |
| Sequences for primers used for RT-qPCR | | |
| PTPRH | Forward Sequence | GAGACGTGGTACAAAGTGGAGG |
|  | Reverse Sequence | GGAAGTGATGGTGACTGTGTCTG |
| RBM15 | Forward Sequence | CTTCCCACCTTGTGAGTTCTCC |
|  | Reverse Sequence | CTTCTTGTTCTCATACCTAACTCC |
| IGF2BP1 | Forward Sequence | CTTTGTAGGGCGTCTCATTGGC |
|  | Reverse Sequence | CCTTCACAGTGATGGTCCTCTC |
| IGF2BP2 | Forward Sequence | GTTGGTGCCATCATCGGAAAGG |
|  | Reverse Sequence | TGGATGGTGACAGGCTTCTCTG |
| IGF2BP3 | Forward Sequence | TCGTGACCAGACACCTGATGAG |
|  | Reverse Sequence | GGTGCTGCTTTACCTGAGTCAG |
| YTHDF2 | Forward Sequence | TAGCCAGCTACAAGCACACCAC |
|  | Reverse Sequence | CAACCGTTGCTGCAGTCTGTGT |
| YTHDF3 | Forward Sequence | GCTACTTTCAAGCATACCACCTC |
|  | Reverse Sequence | ACAGGACATCTTCATACGGTTATTG |
| YTHDC2 | Forward Sequence | GAAAGCTCCTGAACCTCCACCA |
|  | Reverse Sequence | GGTTCTACTGGCAAGTCAGCCA |
| HNRNPA2B1 | Forward Sequence | CAGCAACCTTCTAACTACGGTCC |
|  | Reverse Sequence | CACTGCCTCCTGGACCATAGTT |
| GAPDH | Forward Sequence | CACATCGCTCAGACACCATG |
|  | Reverse Sequence | TTGAGGTCAATGAAGGGGTC |
| β-actin | Forward Sequence | CACCATTGGCAATGAGCGGTTC |
|  | Reverse Sequence | AGGTCTTTGCGGATGTCCACGT |

**Table S1.** Sequences for shRNAs, siRNAs, and primers used for RT-qPCR.


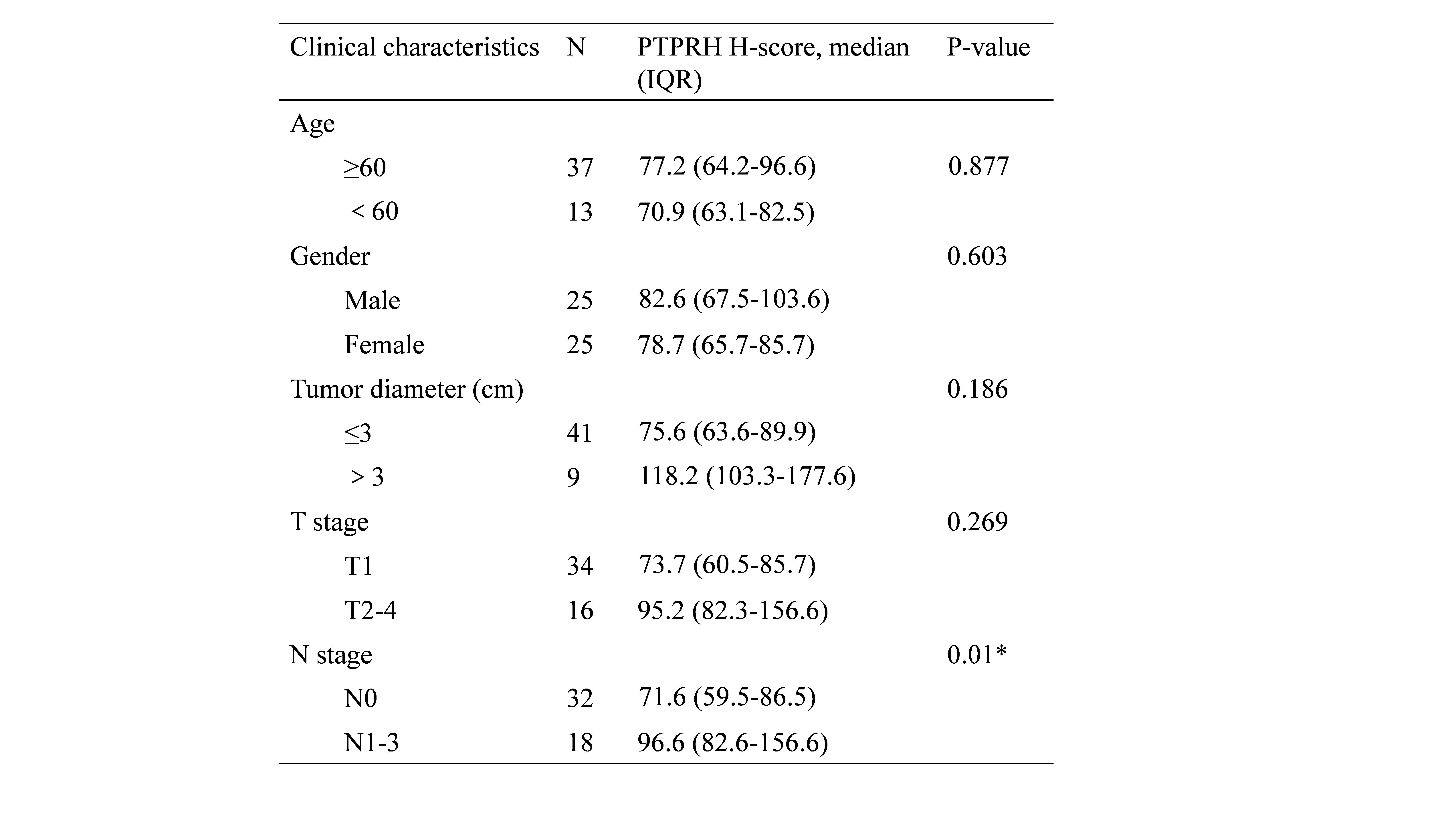


**Table S2.** Clinical-pathological parameters associated with PTPRH protein levels.

**Table S3.** Mass spectrometry analysis of proteins captured by the PTPRH-specific probe.

**Table S4.** The m6A peaks in the RBM15 overexpression group compared to the Negative control group (|log2 FC|≥1.0, P≤0.01).

**Table S5.** Detailed statistical information
