# Supplementary figures and images for: RBM15/IGF2BP2–PTPRH m6A regulatory axis in non-small cell lung cancer
Source: Cell Oncol (Dordr). 2026 May 11;49(3):85. doi: 10.1007/s13402-026-01217-2 (PMC13250017; doi:10.1007/s13402-026-01217-2)

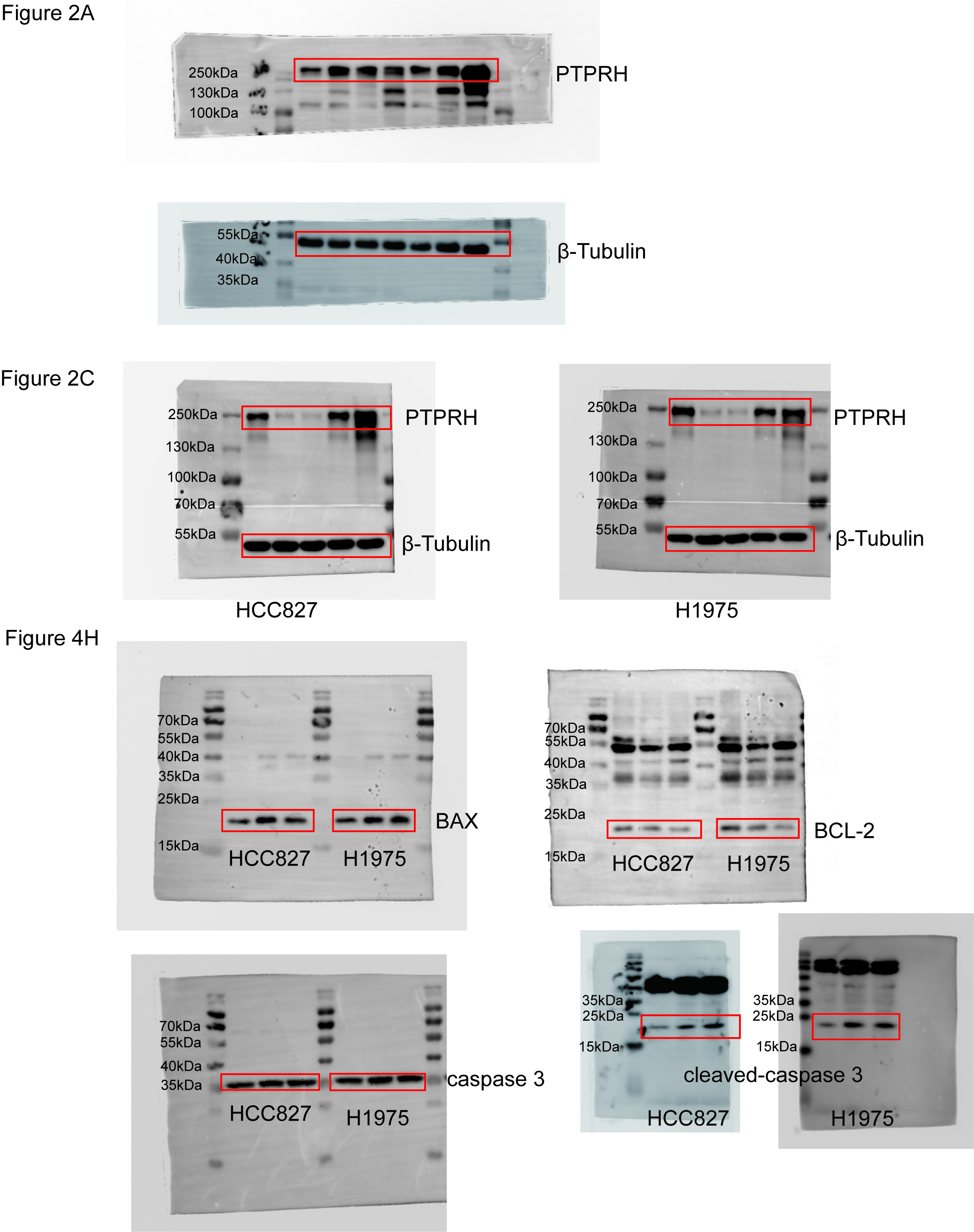

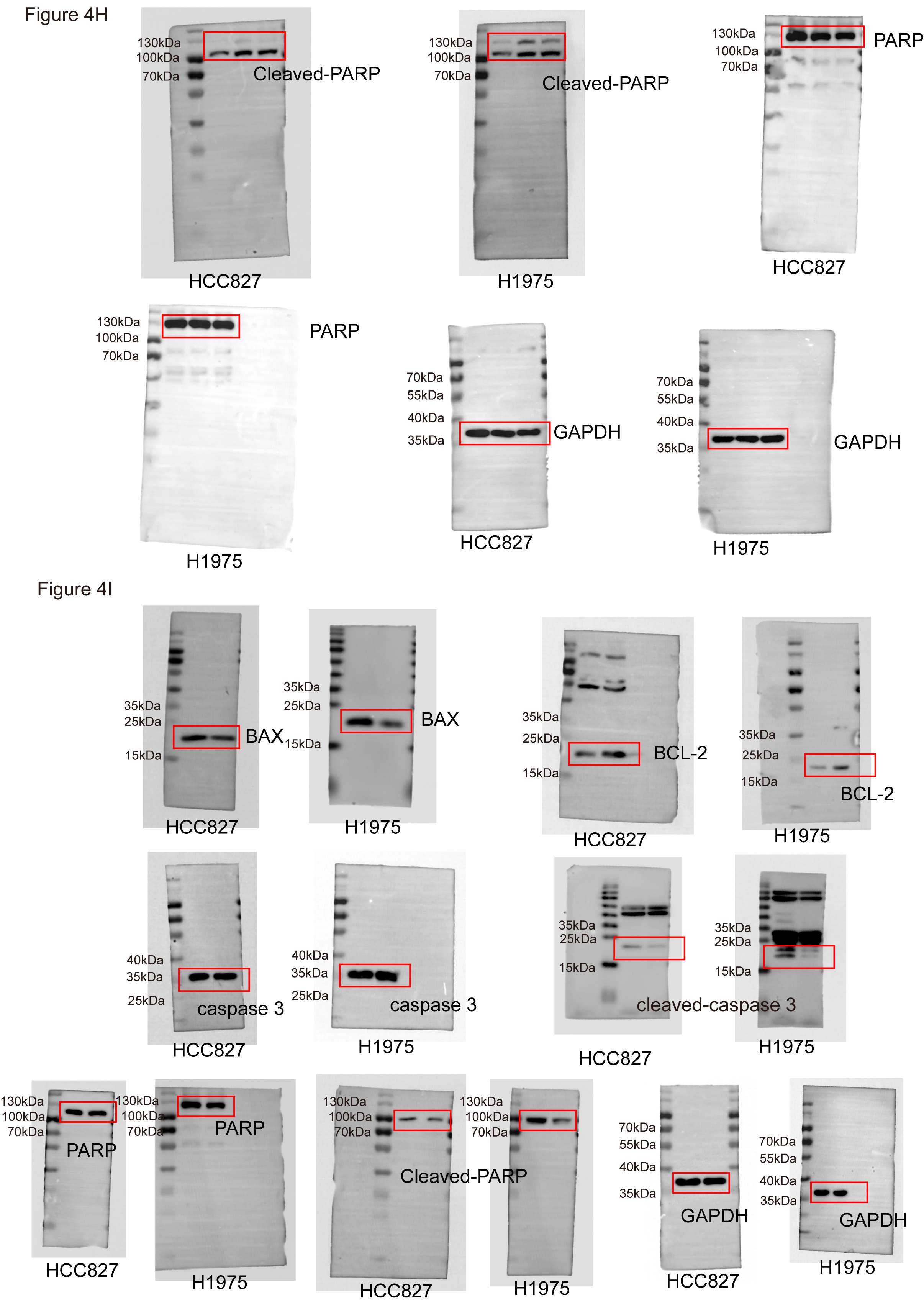

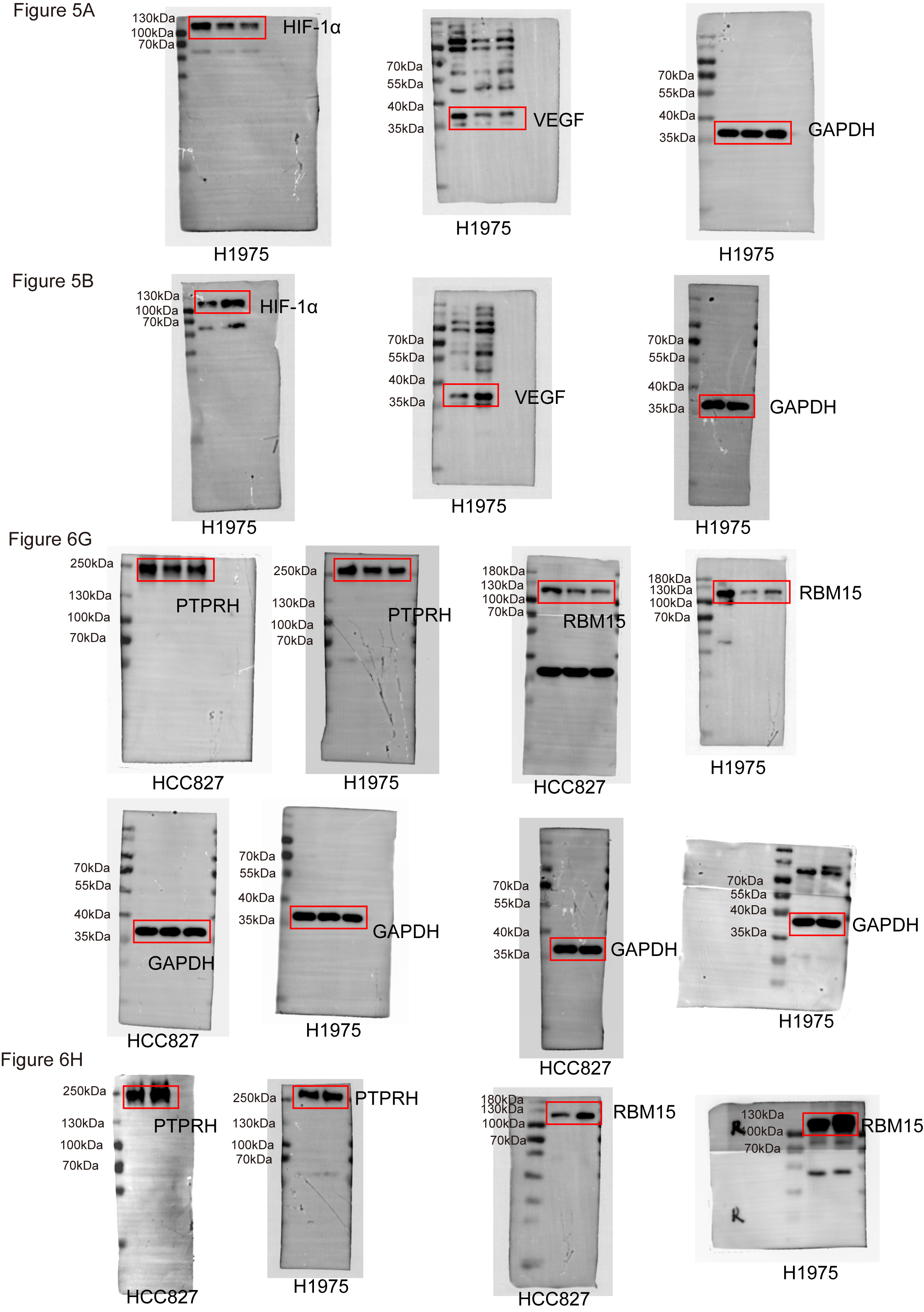

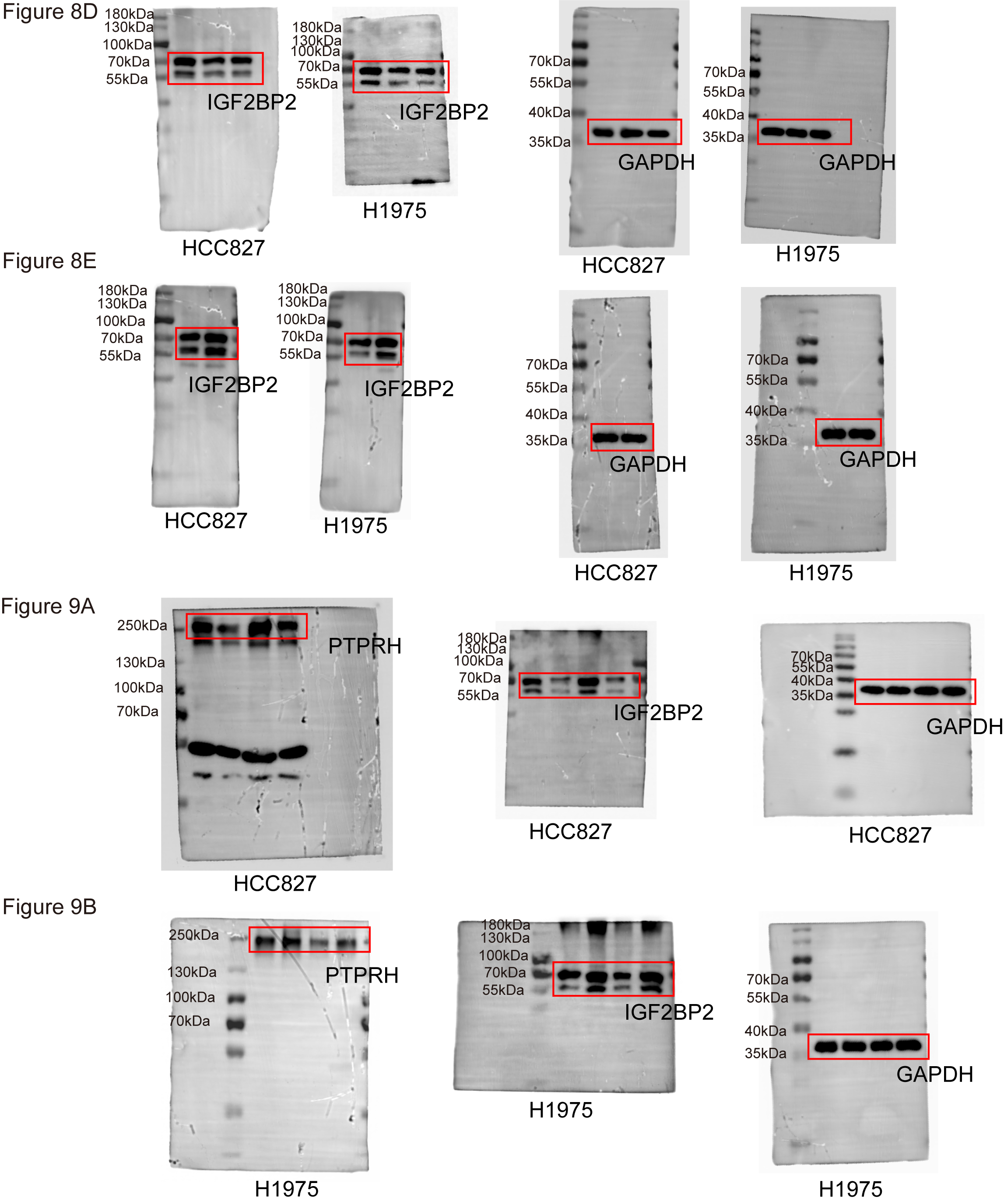


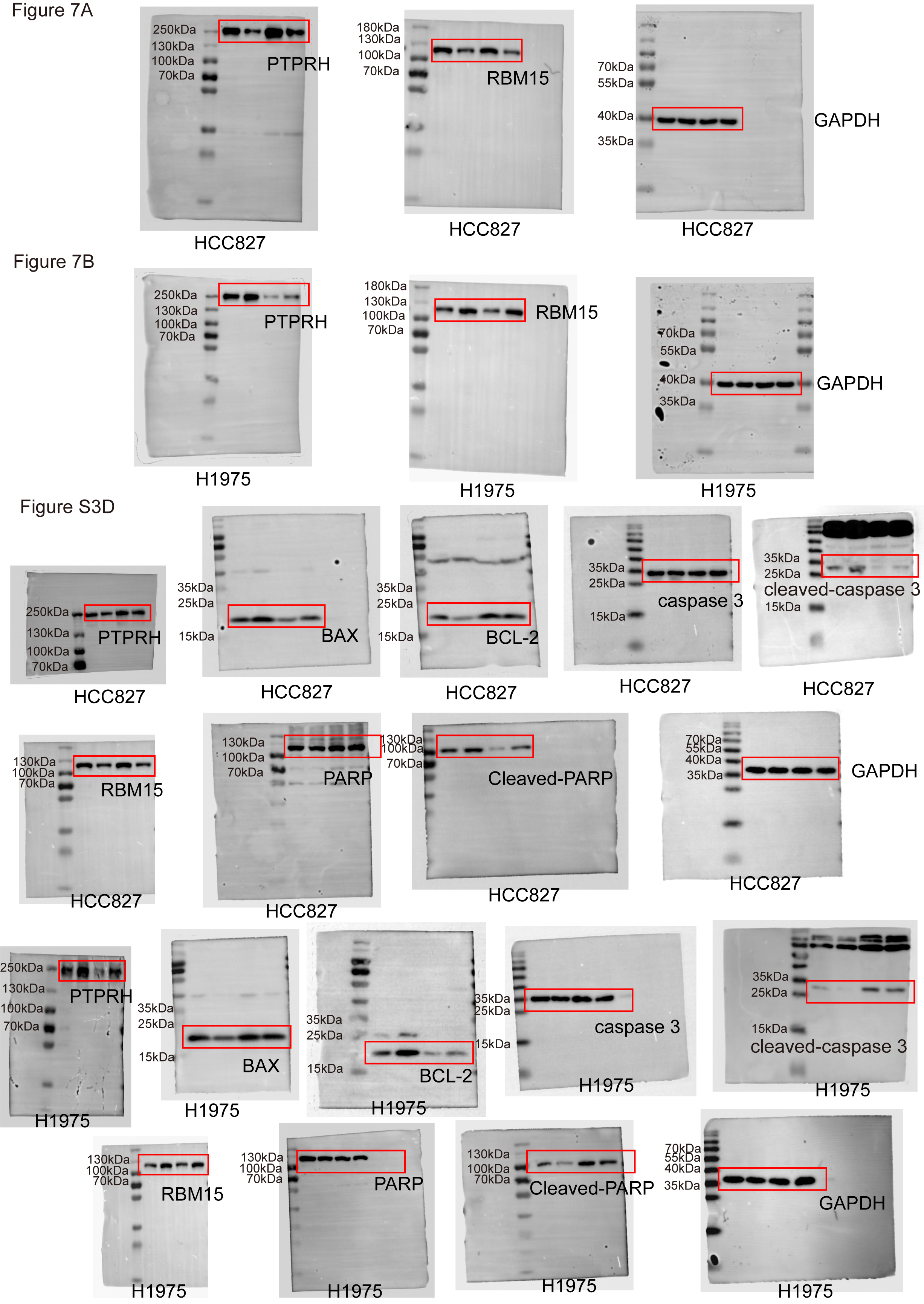

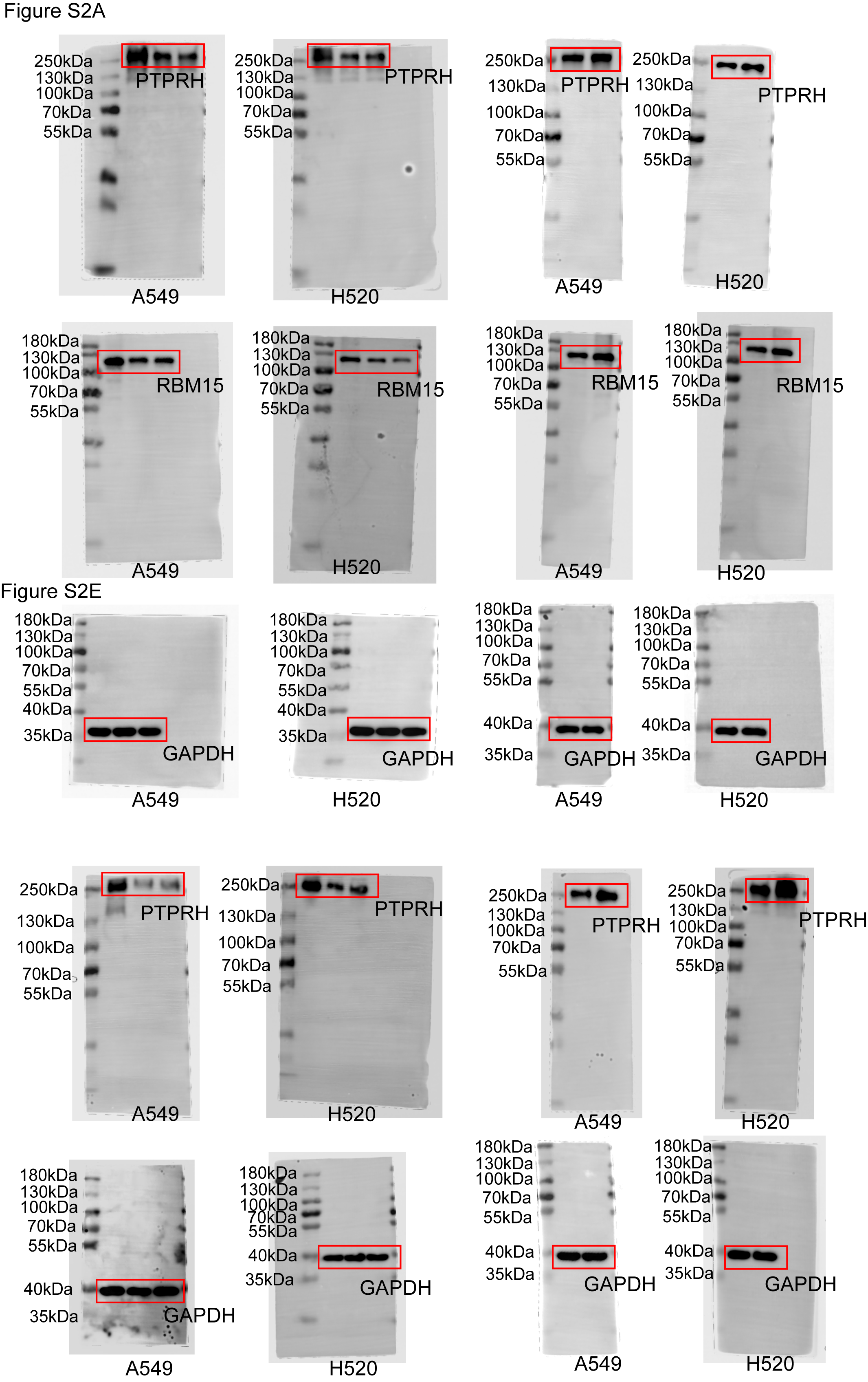

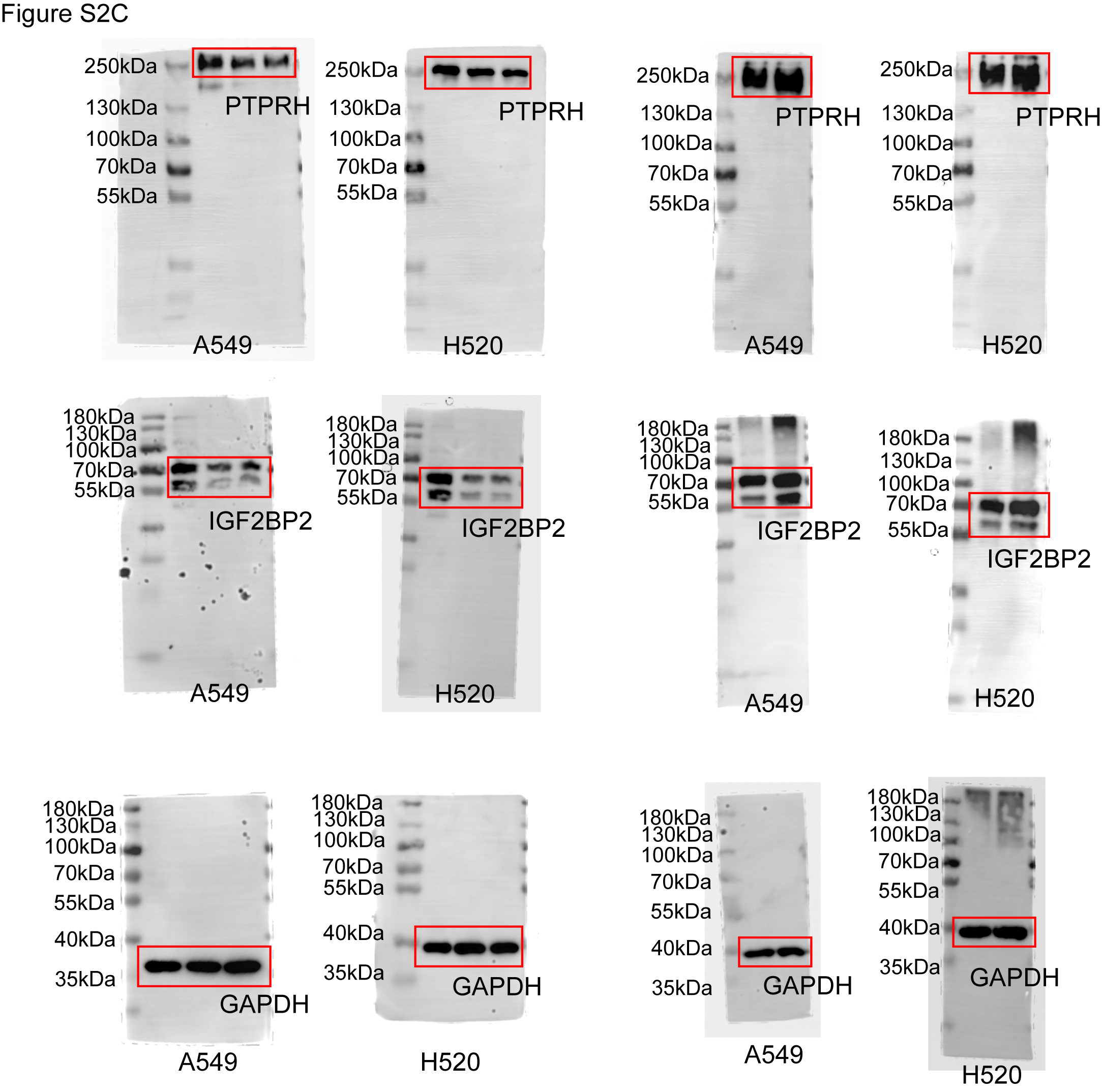

Supplement: Supplementary file 5 — Supplementary material 5 [file 13402_2026_1217_MOESM5_ESM.docx]
